# Competing dynamics of intramolecular deactivation and bimolecular charge transfer processes in luminescent Fe(iii) N-heterocyclic carbene complexes†

**DOI:** 10.1039/d2sc05357h

**Published:** 2023-03-07

**Authors:** Nils W. Rosemann, Linnea Lindh, Iria Bolaño Losada, Simon Kaufhold, Om Prakash, Aleksandra Ilic, Jesper Schwarz, Kenneth Wärnmark, Pavel Chábera, Arkady Yartsev, Petter Persson

**Affiliations:** a Light Technology Institute, Karlsruhe Institute of Technology Engesserstraße 13 DE-76131 Karlsruhe Germany; b Department of Chemical Physics, Lund University Box 124 SE-22100 Lund Sweden arkady.yartsev@chemphys.lu.se; c Department of Theoretical Chemistry, Lund University Box 124 SE-22100 Lund Sweden petter.persson@teokem.lu.se; d Centre for Analysis and Synthesis, Department of Chemistry, Lund University Box 124 SE-22100 Lund Sweden

## Abstract

Steady state and ultrafast spectroscopy on [Fe^III^(phtmeimb)_2_]PF_6_ (phtmeimb = phenyl(tris(3-methylimidazol-2-ylidene))borate) was performed over a broad range of temperatures. The intramolecular deactivation dynamics of the luminescent doublet ligand-to-metal charge-transfer (^2^LMCT) state was established based on Arrhenius analysis, indicating the direct deactivation of the ^2^LMCT state to the doublet ground state as a key limitation to the lifetime. In selected solvent environments photoinduced disproportionation generating short-lived Fe(iv) and Fe(ii) complex pairs that subsequently undergo bimolecular recombination was observed. The forward charge separation process is found to be temperature-independent with a rate of ∼1 ps^−1^. Subsequent charge recombination takes place in the inverted Marcus region with an effective barrier of 60 meV (483 cm^−1^). Overall, the photoinduced intermolecular charge separation efficiently outcompetes the intramolecular deactivation over a broad range of temperatures, highlighting the potential of [Fe^III^(phtmeimb)_2_]PF_6_ to perform photocatalytic bimolecular reactions.

## Abstract 2

Deactivation of an excited Fe^III^ based dye has been investigated. Intramolecular deactivation is comparable to organic dyes, and ultrafast intermolecular deactivation *via* photoinduced disproportionation is observed over a range of low temperatures.

## Graphical abstract

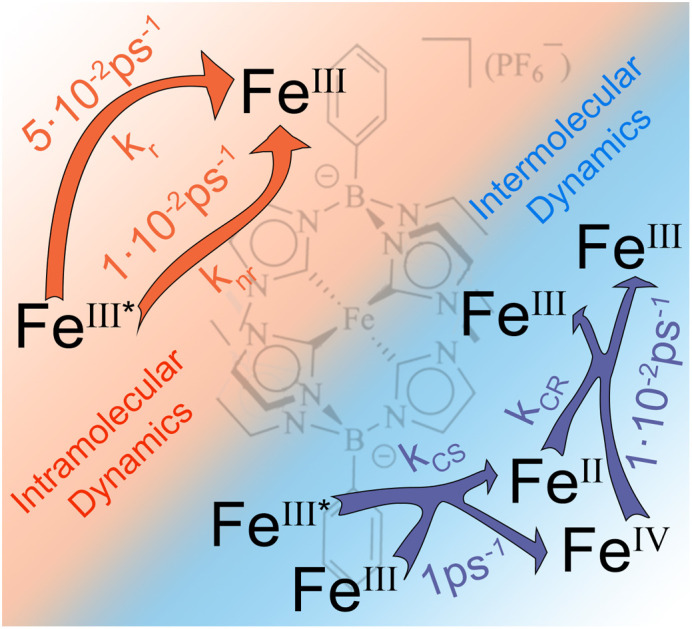

## Introduction

**Scheme 1:**
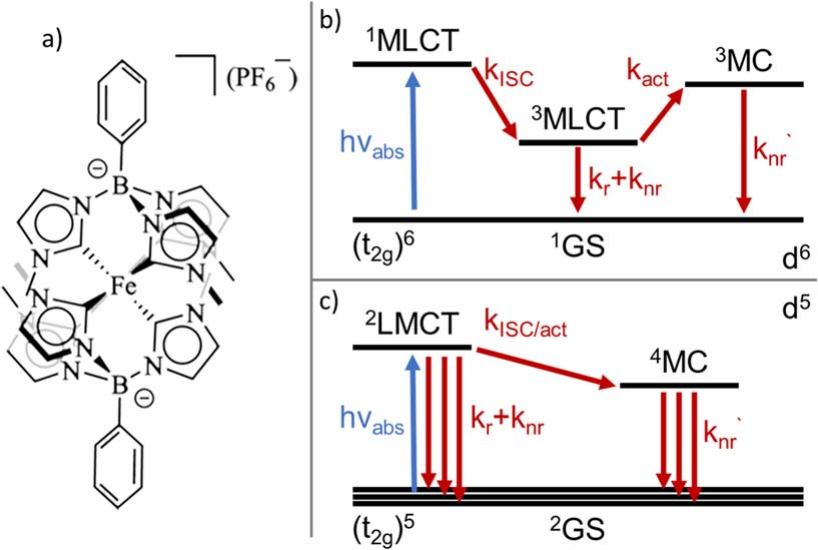
(a) Chemical structure of the [Fe^III^(phtmeimb)_2_]PF_6_ complex. (b) General excited state deactivation schemes for d^6^ light-harvesting complexes. (c) General excited state deactivation schemes for d^5^ light-harvesting complexes.

A variety of earth-abundant photoactive transition metal complexes are currently investigated for emerging sustainable applications such as light-emitting materials, molecular photovoltaics and photocatalysis.^1–3^ The introduction of Fe(iii) N-heterocyclic carbene (NHC) complexes has provided earth abundant photofunctional complexes with dramatically increased excited state lifetimes.^4,5^ This far, the complex [Fe^III^(phtmeimb)_2_]PF_6_ (phtmeimb = phenyl(tris(3-methylimidazol-2-ylidene))borate) (Scheme 1) that shows room temperature photoluminescence and an excited state lifetime of 2 ns is the most promising.^6^ This low-spin 3d^5^ complex features favourable excited state properties involving excitations from a doublet ground state (^2^GS) into photoactive doublet ligand-to-metal charge-transfer (^2^LMCT) excited states. The advantageous excited state redox potentials have allowed such complexes to be used in bimolecular electron transfer reactions including photocatalysis.^7–10^ In contrast, only a very limited number of d^5^ complexes of transition metals *e.g.* Re(ii) and Tc(ii) have previously shown promising photochemical properties.^11–13^ This makes it imperative to understand more generally how the beneficial photophysical and photochemical properties of d^5^ complexes arise.^14,15^ The widespread success of Ru(ii) and other octahedral low spin (singlet ground state) d^6^ complexes for photochemical applications largely relies on ultrafast intersystem crossing (ISC) of the initially excited singlet metal-to-ligand charge-transfer (^1^MLCT) state to a long-lived triplet (^3^MLCT) state.^16,17^ The excited state deactivation pathways from such photoactive ^3^MLCT states have been the subject of many investigations detailing decay channels proceeding either *via* direct but spin-forbidden deactivation to the ^1^GS or activated but spin-allowed decay *via* intermediate triplet metal centred (^3^MC) states (Scheme 1).^18–20^

The deactivation from ^2^LMCT states in Fe(iii) and other d^5^ complexes is intrinsically different to the d^6^ case as the direct deactivation to the ^2^GS is spin-allowed. Furthermore, the lowest accessible MC states are quartets (^4^MC), so that the ^2^LMCT to ^4^MC transition is spin-forbidden (Scheme 1).^14^ To this date only basic information on the deactivation of ^2^LMCT excited states has been obtained.^21^ For example, low-temperature PL data show indications of only moderate overall temperature dependence for the Fe(iii)–NHC complexes.^4,6^ The level of understanding is, however, much less developed compared to that of deactivation pathways in d^6^ complexes. For example, while the d^5^ metal complexes still retain the clear CT character of the excited state, the spin-allowed direct deactivation channel with its typical lifetimes in the 1–10 ns range is in some sense more similar to many organic chromophores^22,23^ including some open-shell (doublet) organic radicals.^24^ Additionally, the ^2^LMCT excited state does not automatically imply long lifetime or strong photoluminescence.^4,25^

Thus, discriminating between the importance of direct and activated channels as the main limiting factor of the excited state lifetime for photofunctional ^2^LMCT excited states in d^5^ complexes becomes important.

Here, we present a thorough spectroscopic and Arrhenius-type analysis of [Fe^III^(phtmeimb)_2_]^+^ (Scheme 1) in order to elucidate the deactivation mechanism governing the excited state lifetime and photoluminescence of this complex.^26–29^ Furthermore, we explore a propensity of [Fe^III^(phtmeimb)_2_]^+^ complexes to undergo photoinduced disproportionation.^30^ Similar to other reports on diiron and Ni^II^ based systems, this process is capable of generating two distinct, and potentially both catalytically active, species from just one type of substrate.^31,32^

Overall, this study provides a new level of understanding of both intra- and intermolecular deactivation mechanisms of Fe(iii)–NHC complexes, and highlights potential routes for further improving this class of sustainable dyes.

## Results and discussion

### Steady-state spectroscopy

As a first step towards distinguishing non-activated and activated decay channels of [Fe^III^(phtmeimb)_2_]PF_6_ we performed steady-state spectroscopy at different temperatures in the range from 80 K to 300 K. [Fe^III^(phtmeimb)_2_]PF_6_ was synthesized as described in the literature.^6^ Measurements were performed in butyronitrile (PrCN), propionitrile (EtCN), 1-propanol, 2-propanol, and a mixture of methanol (MeOH) and ethanol (EtOH) (ratio 1 : 4 by volume detailed information on the solvents and sample mixing is given in the ESI).† In general, alcohols are better suited to perform experiments at low temperatures. They exhibit low freezing points (162 K for MeOH : EtOH and 147 K for 1-propanol) and easily undergo glass transition when cooled appropriately.^33^ Due to the rather high polarity of the alcohols (see Table S2†) the solubility of [Fe^III^(phtmeimb)_2_]PF_6_ is low and spectroscopy can only be performed at low concentrations.^34^ We therefore refer to these solvents as low solubility systems. The nitriles have similar or slightly higher freezing points (161 K for PrCN and 170 K for EtCN) and allow for higher concentrations of [Fe^III^(phtmeimb)_2_]PF_6_ to be achieved easily due to their significantly lower polarity (see Table S2†).^34^ Those solvents will be denoted as high solubility systems. However, the nitriles tend to be unstable when cooled below the glass transition, *i.e.*, a formed glass easily gets chapped and renders spectroscopic experiments impossible.

**Fig. 1:**
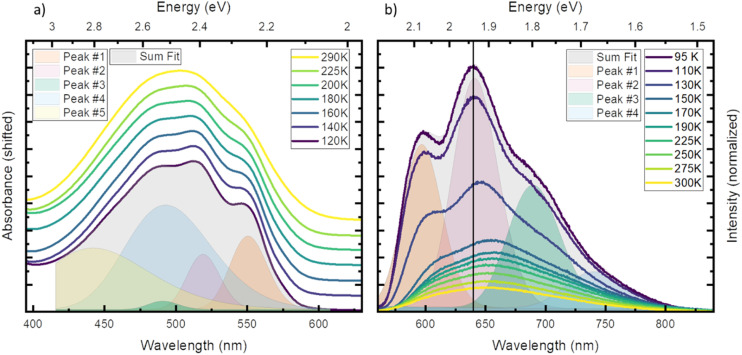
Steady state absorption (a) and emission (b) in PrCN at different temperatures. (a) For comparability the spectra are normalized to the maximum and shifted vertically. (b) Emission spectra after excitation at 510 nm, drawn to scale. Both panels include the fit performed in energy scale of respective spectrum at the lowest measured temperature (grey area) and the individual Gaussian peaks (coloured areas).

First, we investigated temperature dependent steady state absorption. Absorption spectra associated with the ^2^GS to ^2^LMCT transition in [Fe^III^(phtmeimb)_2_]PF_6_ dissolved in PrCN are plotted in Fig. 1 (results for the MeOH : EtOH mixture are shown in Fig. S4†). At low temperatures, the broad absorption peak becomes more structured. The shoulder at 550 nm (2.25 eV) develops into a distinguishable peak and the main peak at ∼500 nm (2.48 eV) splits into sub-peaks. Overall, the peak positions do not vary with temperature only their width and overlap decrease. Additionally, the shape of the absorption spectrum and the temperature dependence are virtually unaffected by the different solvents (see Fig. S1 and S2†).

To gain more insight into the peak structure, we fit the absorption spectra with a series of Gaussian peaks. The best reconstruction of the measured spectra was achieved by a combination of five peaks with the first three peaks sharing the same width and an equal spacing of ∼140 meV (1130 cm^−1^). Thus, we tentatively attribute them to a vibrational progression of the electronic transition from the ground state to the lowest energy excited state. The remaining 2 peaks are best fitted without any constraints and cannot be attributed to the same progression. In our tentative assignment they are taken to represent electronic transitions from the ground state to higher excited states that partially overlap with the vibrational progression of the lowest excited state. The choice of model for the fit is not unambiguous as other models can also fit the resulting spectrum, but a single vibrational progression model is not sufficient (see Fig. S3†). The chosen model, however, yields some similarity of the vibrational progression found in the absorption to the one found in the emission and is affirmed by calculations (*vide infra*). The fit results at the lowest temperature are then taken as input for the higher temperatures, yielding insight into the temperature dependent evolution of the single peaks (see Tables S3 and S4† for full table of the fits results). The series confirms that all apparent changes are caused by increased broadening of the underlying peaks and redistribution of the intensities rather than shifting of the peak energies.

Next, we investigated temperature dependent steady-state emission. The emission spectra of [Fe^III^(phtmeimb)_2_]PF_6_ in PrCN are given in Fig. 1 (results for the MeOH : EtOH mixture are shown in Fig. S5†).

Similar to the absorption, the initially broad emission peak develops into defined sub-peaks when cooled down. However, in contrast to the absorption measurements the peak positions appear to be temperature dependent. To quantify this observation, we fit the emission data at each temperature by the least possible sum of Gaussian peaks. This deconvolution is exemplified in Fig. 1 (respective Fig. S6†) at the lowest temperature (95 K). The best results were obtained using 4 individual peaks with shared width (see Tables S5 and S6† for full tables of the fits results). Even though no condition on the spacing of the peaks was invoked, the best results are obtained for equal spacing between neighbouring peaks. The spacing of ∼140 meV (1130 cm^−1^) is nearly identical to the spacing observed in absorption and is independent of the solvent system. This further strengthens the assignment of the peaks to a vibrational progression. Additionally, a Franck–Condon type analysis of the emission at low temperature was performed (see ESI† for detailed information). Similar to the fit of individual Gaussian peaks, the peak positions and widths are well reproduced when a model with two vibronic energies of 146 meV (1177 cm^−1^) and 90 meV (725 cm^−1^) (see Fig. S9†) is applied. However, the analysis does not provide Huang–Rhys factors that accurately reproduce the spectral intensity distribution. This provides an indication that the emission spectrum contains complicating contributions for example from a vibronic progression or multi-state effects that go beyond the description by a simple Franck–Condon model involving only one electronic transition.

From the measured IR-spectrum of [Fe^III^(phtmeimb)_2_]PF_6_, we find intense peaks around 1100 cm^−1^ (Fig. S24†). The calculated IR-spectrum shows generally good agreement with the measured spectrum, and displays vibrations around 1200 cm^−1^ that involve B–C stretching (see Table S10†) which may contribute to the vibrational features seen in absorption and emission spectra.

In contrast to the ∼140 meV spacing, the shared width of the emission peaks is influenced by temperature (see Fig. S8†). The width increases from 57 meV (460 cm^−1^) at 95 K to 72 meV (581 cm^−1^) at 300 K. We assign this effect to inhomogeneous broadening, where the dissolved molecules experience a larger possibility of solvent arrangements as the temperature increases.^35^

In stark contrast to the absorption, the emission peaks undergo a temperature dependent shift (see Fig. S7†). In both solvent systems, an emission blue shift by ∼50 meV is observed when heating the system. In general, the shift of emission energy and the virtually constant absorption peaks indicate that the system undergoes reorientation and relaxation after excitation. This relaxation is hindered below the freezing point of the solvent as the frozen solvent cannot rearrange around the excited molecule and the system cannot relax. Furthermore, some vibrational modes may be hindered or are no longer accessible for non-radiative decay when the solvent is frozen;^28^ resulting in increased luminescence intensity that is also observed in Fig. 1.

**Fig. 2:**
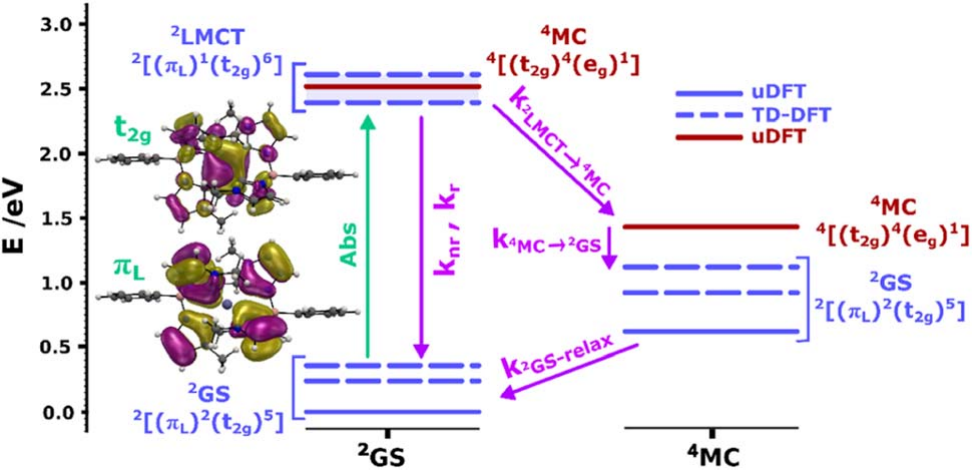
Calculated doublet (blue bars) and quartet (red bars) ground and excited states for the [Fe^III^(phtmeimb)_2_]^+^ complex at the ^2^GS (left column) and ^4^MC (right column) geometries. Dashed bars correspond to TD-DFT results and bold bars to uDFT results. The multiple ^2^LMCT states are represented as a band in blue shadow. Molecular orbital representations of the electron transition in the lowest ^2^LMCT state are also presented (full-size MO figures available in the ESI†). Calculations were performed at the B3LYP (12% Hartree–Fock exchange)/def2-TZVP/PCM(acetonitrile) level of theory.

To support our assignment of the transitions involved in absorption and emission, we performed TDDFT calculations on the optimized [Fe^III^(phtmeimb)_2_]^+^ structure. The calculations reveal three quasi-degenerate excited states with similar LMCT nature (see Fig. 2), consistent with general expectations for this type of d^5^ complex.^14^ This highlights that absorption indeed can occur from the ^2^GS to more than one excited electronic state. Calculated oscillator strengths indicate that higher energy transitions are favourable in absorption (see Table S15†). This suggests that absorption is followed by sequential relaxation of the molecule and internal conversion to the lowest lying excited state if Kasha's rule is met. The optimized geometry of the ^2^LMCT state was also found to differ from the ^2^GS state (Table S13†), meaning that the excited state potential energy minima should be offset to the ground state. Overall, the results from steady state spectroscopy fit well within the Franck–Condon model with the peculiarity of several closely lying ^2^LMCT-states available for excitation.

### Intramolecular dynamics

To gain insight into the deactivation dynamics we performed time-resolved emission measurements using time-correlated single photon counting (TCSPC). Shown here are results from the high solubility system PrCN. Results for low solubility systems (MeOH : EtOH and 1-propanol) are similar and are shown in the ESI.†

**Fig. 3:**
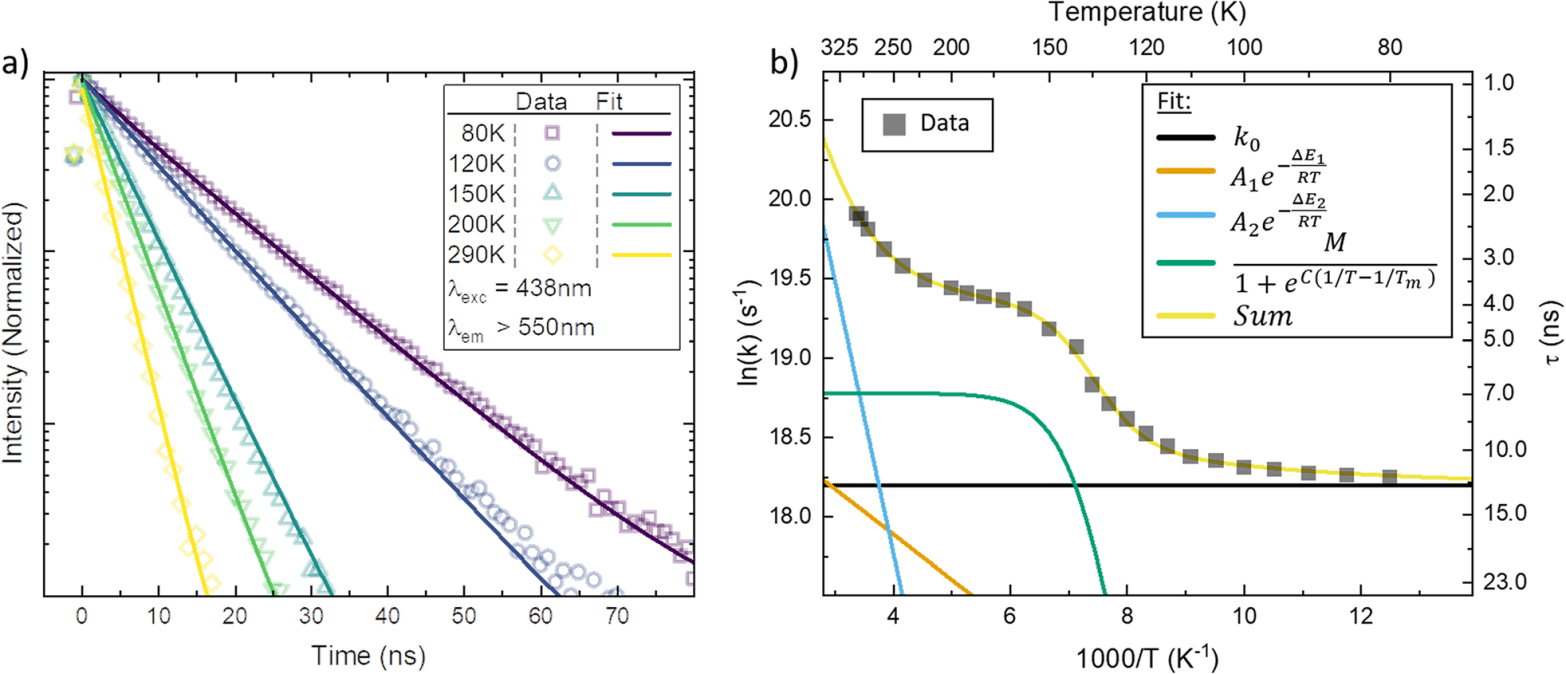
(a) Temperature dependent luminescence decay measured by spectrally integrated time-correlated single photon counting (TCSPC) in PrCN. Data at selected temperatures are shown together with a double exponential fit to the data. (b) The major rate constant fitted to the TCSPC data (black squares) plotted as a function of inverse temperature together with the Arrhenius fit (yellow line) to the data. Additionally, the decomposition of the individual components in the Arrhenius fit is given (coloured lines).

**Fig. 4:**
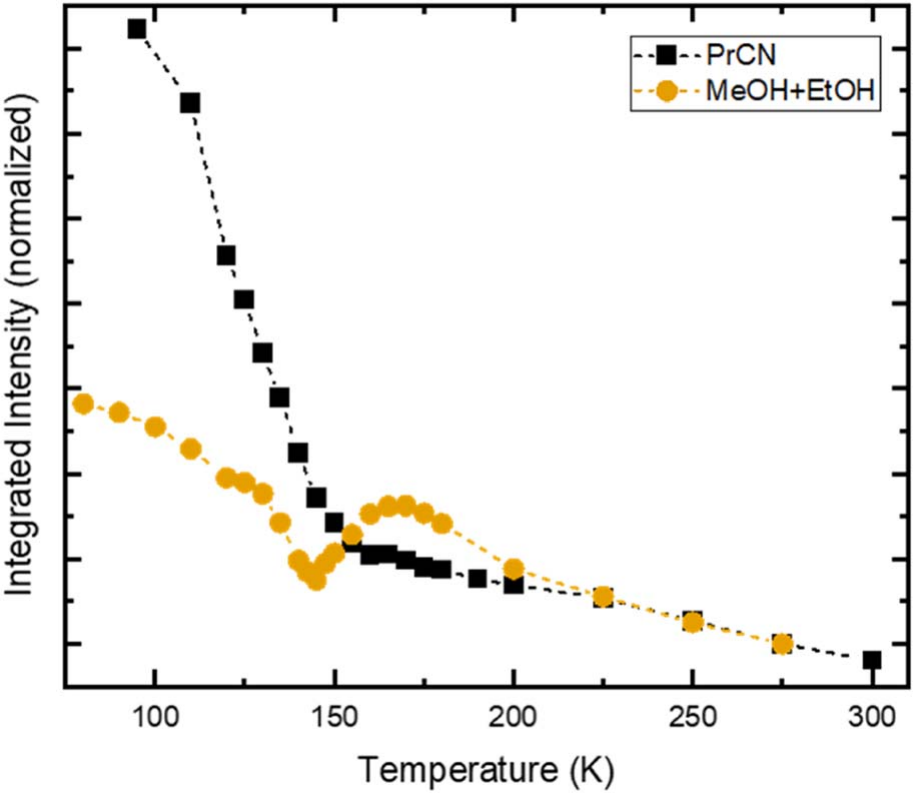
Temperature dependent integrated emission intensity of [Fe^III^(phtmeimb)_2_]PF_6_ in PrCN (black squares) and MeOH : EtOH-mixture (orange circles), following excitation at 500 nm. For comparison, the integrated intensities are normalized to their respective value at 275 K. Dashed lines represent guide to the eye.

In Fig. 3a, the emission transients of [Fe^III^(phtmeimb)_2_]PF_6_ in PrCN are systematically prolonged as the temperature is lowered (all measured temperatures in Fig. S18†). Above the solvent freezing point (∼140 K), the emission decay was fitted by a single exponential. Below, a minor second component was required for sufficient fitting of the data over the whole time-range (see Fig. S19†). The temperature dependence of the major decay component is presented in Fig. 3b. The lifetime increases from 2 ns at 290 K to 11 ns at 80 K (Table 2). Based on the temperature dependent absorption spectra and the observed emission lifetime we followed the Strickler–Berg formalism to calculate the radiative (*k*_r_) and non-radiative rate (*k*_nr_) as well as the emission quantum yield (*η*); additionally the quantum yield is directly determined from emission intensity measurements (*cf.*Fig. 4). All values are summarized in Table 1 and detailed information on the calculations and measurements is given in the ESI.† The quantum yield increases from ∼3% at 290 K to ∼25% at 80 K. Since the absorption does not show strong temperature dependence the increase in quantum yield suggests that non-radiative decay pathways are blocked by cooling. This is corroborated by the virtually unchanged radiative rate and the strong change in non-radiative rate.

**Table 1:** Rate constants and luminescence quantum yield (*η*) at 300 K and 80 K for [Fe^III^(phtmeimb)_2_]^+^ (this work) in PrCN, [Ru^II^(dqp)_2_]^2+^,^29^ [Ru^II^(pymbpy)_2_]^2+^,^36,37^ and the organic dye Coumarine 47 at 300 K (ref. 38)

Complex	*k*_0_ (s^−1^)	300 K					80 K			
		*k*_r_ (s^−1^)	*k*_nr_ (s^−1^)	*k*_1_ (s^−1^)	*k*_2_ (s^−1^)	*η*	*k*_r_ (s^−1^)	*k*_nr_ (s^−1^)	*k*_1_ (s^−1^)	*η*
[Fe^III^(phtmeimb)_2_]^+^	8 × 10^7^	1 × 10^7^	5 × 10^8^	9 × 10^7^	2 × 10^8^	0.02	2 × 10^7^	7 × 10^7^	4 × 10^6^	0.25
[Ru^II^(dqp)_2_]^2+^^a^	1 × 10^5^	6 × 10^3^	3 × 10^5^	9 × 10^4^	5 × 10^4^	0.02	7 × 10^3^	1 × 10^5^	3 × 10^3^	0.06
[Ru^II^(pymbpy)_2_]^2+^^b^	4 × 10^5^	7 × 10^4^	2 × 10^6^	3 × 10^5^	6 × 10^7^	0.001	6 × 10^4^	2 × 10^5^	2 × 10^4^	0.21
Coumarine 47	2 × 10^8^	1 × 10^7^	3 × 10^8^	7 × 10^6^		0.04				

a The abbreviation dqp is short for the ligand 2,6-di(quinolin-8-yl)pyridine.^29^

b The abbreviation pymbpy is short for the ligand 6-(2-picolyl)-2,2′-bipyridine.^36,37^

As suggested by the standard model in Fig. 2, the deactivation of the ^2^LMCT state(s) can essentially occur *via* radiative and non-radiative decay directly to the ^2^GS manifold, or by a thermally activated non-radiative pathway *via*^4^MC states. To test this model, we perform an Arrhenius type analysis on the data plotted in Fig. 3b. The data are best fitted using an Arrhenius model with four components given by:^17^
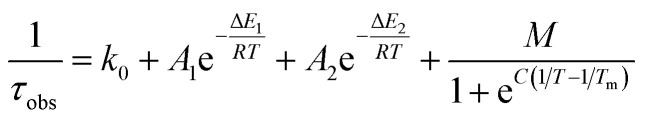
where *τ*_obs_ is the observed temperature-dependent luminescence lifetime, *k*_0_ reflects the sum of the temperature-independent radiative and non-radiative decay rates from ^2^LMCT to ^2^GS, *A*_1_ and Δ*E*_1_ are pre-exponential factors and activation energy for the first Arrhenius term, and *A*_2_ and Δ*E*_2_ for the second Arrhenius term, respectively. The *M*-term is an empirical term that accounts for changes to the non-radiative rate from translational and rotational degrees of freedom occurring at the glass transition of the solvent rather than any activated intrinsic molecular process. Here *T*_m_ is the glass transition temperature of the solvent, and *M* and *C* are fitting parameters used to describe the smoothness of the phase-transition.

The results of the Arrhenius analysis are collected in Table 2. Additionally, the relative significance of the four individual components are plotted as a function of temperature in Fig. 3b. As seen in Table 2 and Fig. S20,† the analysis is qualitatively similar in all investigated solvents, with the main difference being the glass transition temperature. Overall, the dynamics of emission decay appear to be largely independent of the solvent, indicating that the decay is governed by the inherent properties of [Fe^III^(phtmeimb)_2_]PF_6_.

**Table 2:** Lifetimes (at 100 K and 300 K) and Arrhenius parameters for [Fe^III^(phtmeimb)_2_]^+^ (this work) in different solvent systems, [Ru^II^ (pymbpy)_2_]^2+^,^36,37^ [Ru^II^ (dqp)_2_]^2+^,^29^ and Coumarine 47 (ref. 22)

Complex	*τ* 100 K (ns)	*τ* 300 K (ns)	*k*_0_ (s^−1^)	*M* (s^−1^)	*C* (K)	*T*_m_ (K)	*A*_1_ (s^−1^)	*E*_1_ (cm^−1^)	*A*_2_ (s^−1^)	*E*_2_ (cm^−1^)
[Fe^III^(phtmeimb)_2_]^+^ (PrCN)	11	2	8.0 × 10^7^	1.4 × 10^8^	2280	139	1.8 × 10^8^	200	4.9 × 10^10^	1190
[Fe^III^(phtmeimb)_2_]^+^ (1-propanol)	11	2	9.0 × 10^7^	1.9 × 10^8^	2040	183	1.4 × 10^8^	200	4.8 × 10^10^	1190
[Fe^III^(phtmeimb)_2_]^+^ (MeOH : EtOH)	10	2	9.0 × 10^7^	2.1 × 10^8^	2350	146	1.5 × 10^8^	180	5.1 × 10^10^	1200
[Ru^II^(pymbpy)_2_]^2+^	3700	17	3.9 × 10^5^	6.9 × 10^5^	3700	115	4.4 × 10^5^	100	1.0 × 10^15^	3400
[Ru^II^(dqp)_2_]^2+^	8500	3000	1.2 × 10^5^	9.9 × 10^4^	6200	119	3.0 × 10^5^	260	1.5 × 10^10^	2600
Coumarine 47	5^a^	4	2.0 × 10^8^	—	—	—	4.3 × 10^9^	850	—	—

a Lifetime at 115 K.

According to the Arrhenius model, the measured emission rate is predicted to reach a plateau of 1/13 ns^−1^ (Fig. 3b) when all temperature-activated decay pathways are frozen out. At 80 K, the relative emission quantum yield is ∼25% implying that *k*_0_ is roughly 4 times higher than the radiative rate *k*_r_ (Table 1). Noticeably, above 120 K we observe a large increase in the non-radiative deactivation rate (green line in Fig. 3b) that is not associated with an activated process. At this temperature range, the solvent undergoes a transition from solid to liquid. Below 120 K the molecules experience a rigid environment, that impedes reorganization and blocks structural degrees of freedom. The additional non-radiative deactivation channel above 120 K implies that less restricted vibrations in the molecule or solvent reorganization contribute significantly to additional non-radiative decay in solution. The red-shift of emission above the melting temperature discussed above is most probably related to the same effect and indicates the possibility of an additional structural reorganization or solvent reorganization following excitation.

The first Arrhenius term (*k*_1_) with Δ*E*_1_ of 200 cm^−1^ (24.7 meV) (see Table 2) contributes already from 80 K. Due to the relatively small pre-exponential factor (*A*_1_ ∼ 10^8^ s^−1^), the contribution of this temperature-activated term never exceeds that of the *k*_0_-term in the observed temperature range. The second Arrhenius term (*k*_2_) has a higher activation energy of Δ*E*_2_ 1190 cm^−1^ (147.5 meV) and thus starts to contribute significantly only above ∼200 K. Compared to *A*_1_ the pre-exponential factor *A*_2_ (∼10^11^ s^−1^) is three orders of magnitude larger. Hence, already from ∼250 K the second activated term has a higher contribution than *k*_0_ and eventually outcompetes all other terms. Nevertheless, all 4 processes contribute significantly to the non-radiative decay rate: the intrinsic deactivation expressed by *k*_0_ together with the empirical term is largest (50%), followed by the activated terms *k*_2_ (35%) and *k*_1_ (15%).

In analogy to previous studies on Ru(ii)-polypyridyl type d^6^/MLCT complexes, we associate the term *k*_1_ with the activation of the lowest excited CT state population to efficient channels of internal conversion to the ground state. The term *k*_2_ is, in contrast, conventionally associated with the activated transition from the excited CT to the MC manifold of states.^17^ In our case, the involved states are the lowest energy ^2^LMCT and ^4^MC states (see Fig. 2), with corresponding ^2^MC states expected to be higher in energy.

To put our observations in perspective, here we compare [Fe^III^(phtmeimb)_2_]PF_6_ to representative bistridentate complexes such as [Ru^II^(pymbpy)_2_][PF_6_]_2_, [Ru^II^(dqp)_2_][PF_6_]_2_ and an organic dye Coumarine 47, where temperature dependences of the emission have been analysed according to the Arrhenius model (Tables 1 and 2).^22,29,37^ The complex [Ru^II^(pymbpy)_2_][PF_6_]_2_ has a lifetime of 17 ns at 300 K, that is prolonged to ∼4 μs when cooled down. For this complex, the second activated term *k*_2_ clearly dominates the deactivation rate above ∼230 K and drastically reduces the lifetime of this complex, see Fig. S21.† In [Fe^III^(phtmeimb)_2_]PF_6_ and [Ru^II^(dqp)_2_][PF_6_]_2_ the *k*_2_ term has lower activation energies but does not dominate the deactivation that much. This is because of the much smaller pre-exponential factors, which could be due to the high rigidity of the ligands in these complexes making it harder to convert into the distorted ^4^MC and ^3^MC states. This goes in line with the fact that for [Fe^III^(phtmeimb)_2_]PF_6_ and [Ru^II^(dqp)_2_][PF_6_]_2_ the lifetime is actually not very temperature dependent (Table 2), which indicates that the non-activated deactivation pathways are not dominating the decay of these complexes. In [Fe^III^(phtmeimb)_2_]PF_6_ the temperature independent pathways are spin-allowed. In [Ru^II^(dqp)_2_][PF_6_]_2_ and other long-lived Ru(ii)-polypyridyl type complexes, however, the direct deactivation to the ground state is spin-forbidden. Thus, the absence of ISC is the reason why the lifetime of [Fe^III^(phtmeimb)_2_]PF_6_ is much shorter than that of [Ru^II^(dqp)_2_][PF_6_]_2_, and instead similar to that of Coumarine 47. This is reflected in *k*_0_ (Table 2) where [Fe^III^(phtmeimb)_2_]PF_6_ and Coumarine 47 are similar, and the Ru-complexes have much smaller values. The temperature dependence of the emission determined in this study therefore suggests that the simple strategy of shifting the MC manifold upwards in energy from the ^2^LMCT will probably not be sufficient to increase the excited state lifetime of Fe(iii)-carbenes and related complexes much further.

To consider the underlying cause of the efficient non-activated deactivation from ^2^LMCT to ^2^GS, we compare the optimized geometries of both states (Table S11†). The main difference between the ^2^LMCT and ^2^GS lies in the N–B bond lengths on one of the ligands, which structurally rearranges to prolong one and shorten two of the bonds. This highlights the ligand's rigidity around the metal centre and the successful design strategy. However, it also reveals the flexibility in the backbone of the ligand. The flexibility is reflected in vibrational modes in the range of ∼1200 cm^−1^ (see Table S12†). These accessible vibrational modes are a potential pathway for unwanted direct deactivation of the excited state. Hence, our findings indicate that a more rigid design of the outer part of the ligand might favour prolonged lifetime and emission quantum yield.

### Intermolecular dynamics

In stark contrast to the unaffected emission dynamics, we find that the temperature dependent emission intensity is strongly influenced by the solvent (see Fig. S5†). To highlight the difference, the emission intensity *versus* temperature is plotted in Fig. 4 for the two types of solvents. For the case of high solubility (PrCN) the emitted intensity increases slightly upon cooling. Below the glass transition temperature, the intensity increases drastically, similar to what was found in the Arrhenius analysis regarding the emission lifetime. The eight-fold increase in emission intensity also agrees with values calculated based on the Strickler–Berg formalism (*cf.* Fig. S24†). For the low solubility solvent (MeOH : EtOH), the intensity also increases at first. Towards the glass transition temperature, however, the intensity experiences a steep drop. For lower temperatures this drop recovers slightly.

To explain this drop in emission intensity, we compare the temperature dependent transient absorption (TA) spectroscopy data in all studied solvents. The TA spectra in both nitrile solvents (high solubility) do not change significantly over the course of temperatures, and resemble the spectrum first published in acetonitrile (*cf.* Fig. S10†).^6^

**Fig. 5:**
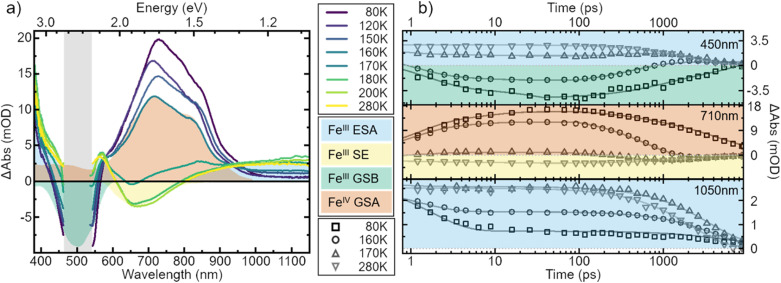
Transient absorption spectra of [Fe^III^(phtmeimb)_2_]PF_6_ in MeOH : EtOH 1 : 4 volume mix at selected temperatures. (a) Spectra recorded 100 ps after excitation at 500 nm. All spectra are corrected for the background signal by subtracting a pre-pulse spectrum, recorded at −20 ps time delay. Additionally, the spectra have been cut to remove excitation scatter (grey area). (b) Kinetics recorded at wavelengths 450 nm, 710 nm and 1050 nm during the course of temperatures correspond to different spectral features with lines representing multi-exponential fits. The ultrafast time evolution has here been omitted. Note that the data are not normalized but shown as measured.

Fig. 5a gives the TA spectra at multiple temperatures in MeOH : EtOH. Strikingly, in all low solubility solvents the spectral signature changes its shape completely with varying temperature (Fig. S11†). A remarkable feature of the TA signature at room temperature is the absence of a negative signal in the ground-state bleach (GSB) region ∼500 nm due to strong excited-state absorption (ESA).^6^ At low temperatures, however, we now observe a negative GSB feature in the low solubility solvents suggesting that ESA in this spectral range has drastically decreased similarly to the infrared ESA. Furthermore, with decreasing temperature, the negative stimulated emission (SE) signature ∼650 nm converts into a positive signal. Comparison of the newly emerged positive broad feature ∼730 nm reveals that it matches perfectly the ground-state absorption of [Fe^IV^(phtmeimb)_2_]2PF_6_, *i.e.*, the oxidized species of the molecule that was proven to be stable in a prior study.^39^ A detailed deconvolution of the TA signature for all investigated solvents is given in Fig. S12 and S13.† We explain the observed changes in signature by the occurrence of charge separation (CS), which is an electron transfer between one excited molecule and a neighbouring molecule in the ground state, forming one oxidized complex, *i.e.*, [Fe^IV^(phtmeimb)_2_][PF_6_]_2_ and one reduced complex, *i.e.*, [Fe^II^(phtmeimb)_2_].

[Fe^III^(phtmeimb)_2_]PF_6_ has been shown to undergo CS upon excitation in solution at high concentrations.^30^ In our case CS is observed at nominally very low concentrations (<0.5 μM). However, CS appears in the temperature range of 140–180 K, which is similar to the temperature range in which the drop in emission intensity occurs (*cf.*Fig. 4) and the freezing point of the solvent (∼160 K). Consequently, we assign the appearance of CS to a temperature dependent aggregation and thus a local increase in concentration of the solute. This is underpinned by the fact that aggregation effects often are prominent in polar solvents such as alcohols and increase at lower temperatures.^22^

In the case of CS, a signal from [Fe^II^(phtmeimb)_2_] should also be observed. [Fe^II^(phtmeimb)_2_] absorbs around 350 nm and thus we measured the TA signal of [Fe^III^(phtmeimb)_2_]PF_6_ in MeOH : EtOH in the UV spectral region 290–380 nm at three selected temperatures. However, the same spectral region features a strong ESA signal of [Fe^III^(phtmeimb)_2_]PF_6_ which naturally decays when [Fe^II^(phtmeimb)_2_] is formed. Nevertheless, we observe clear changes in this spectral region that underline the assignment of CS (see Fig. S15 and S16†).

To strengthen the assignment of CS and to further elucidate this process, we investigate the underlying dynamics. TA kinetics are integrated over significant regions of interest: (i) ∼450 nm where a positive signal corresponds to ESA of the excited Fe^III^-species, while a negative signal corresponds to GSB. (ii) ∼710 nm where a negative signal corresponds to SE of the Fe^III^-species, while a positive signal indicates ground-state absorption (GSA) of the Fe^IV^-species in the absence of the excited Fe^III^-species. (iii) ∼1050 nm where the only expected signal is positive ESA from the excited Fe^III^-species. (iv) ∼350 nm where the GSA of the Fe^II^-species contributes a strong positive signal. However, a positive signal in region (iv) is not unambiguous since Fe^IV^-species and excited Fe^III^-species also contribute a positive signal there. The corresponding kinetics in the regions (i–iii) at a selection of temperatures are plotted in Fig. 5b. In region (i) the signal changes from positive above 170 K to negative at lower temperatures. This indicates a vanishing of excited Fe^III^-species without actual recovery of the Fe^III^-species in the ground state. The signal in region (ii) changes from negative above 170 K to positive at lower temperatures, indicating again a loss of excited Fe^III^-species but accompanied by a gain in ground state Fe^IV^-species. At last, in region (iii) the overall signal intensity decreases for decreasing temperature, again indicating an overall loss in excited Fe^III^-species. Notably, all of the observed signal changes are not instant but appear on timescales that can clearly be resolved in the experiment.

In all solvents we observe processes in two distinct time ranges. For high solubility solvents the ESA is virtually constant while the SE increases slightly (Fig. S14†). We take this as an indication of minor reorganization or relaxation of the excited molecule. The same is observed in the low solubility solvents for temperatures above ∼170 K. For lower temperatures, however, the whole spectral signature changes. Since we attribute these changes to the occurrence of the oxidized and reduced species, we assign the initial fast changes (<100 ps after excitation) to charge separation CS. The subsequent dynamics are then assigned to charge recombination (CR) as well as decay of complexes that did not undergo CS.

Assuming maximum CS at 100 ps, we estimate the number of excited molecules that take part in bimolecular charge transfer reactions. For this, we record the temperature dependent intensity of the ESA feature at 1050 nm that corresponds to the loss of excited Fe^III^-species (Fig. S17†). This comparison reveals a drastic drop between 170 K and 150 K with only 20% of the excited Fe^III^-species population that is observed at the highest temperature remaining observable at low temperatures. This is similar to the observed drop in emission intensity (Fig. 4). Comparing the relative emission intensities at 140 K in both solvent systems (Fig. 4) gives an upper estimation that 40% of the emissive Fe^III^-species do not undergo CS in MeOH : EtOH. The discrepancy between TA and emission measurements can be explained by the different concentrations of [Fe^III^(phtmeimb)_2_]PF_6_. TA measurements in MeOH : EtOH were performed at 430 μM whereas emission measurements in the same solvent used 380 μM. The lower concentration makes aggregation at low temperatures less likely and thus decreases the number of molecules that can undergo CS. Overall, a process like CS will likely depend on sample conditions such as concentration of the solute and impurities. While interesting, an exact quantification of the influence of these conditions is beyond the scope of this report. It is, however, noteworthy that below 150 K the solvent is frozen, consequently no diffusion related processes are possible. Thus, the dynamics of CS that we observe are solely governed by the underlying energetics and not by diffusion, which is in contrast to prior reports on CS in this complex.^7,30^

**Fig. 6:**
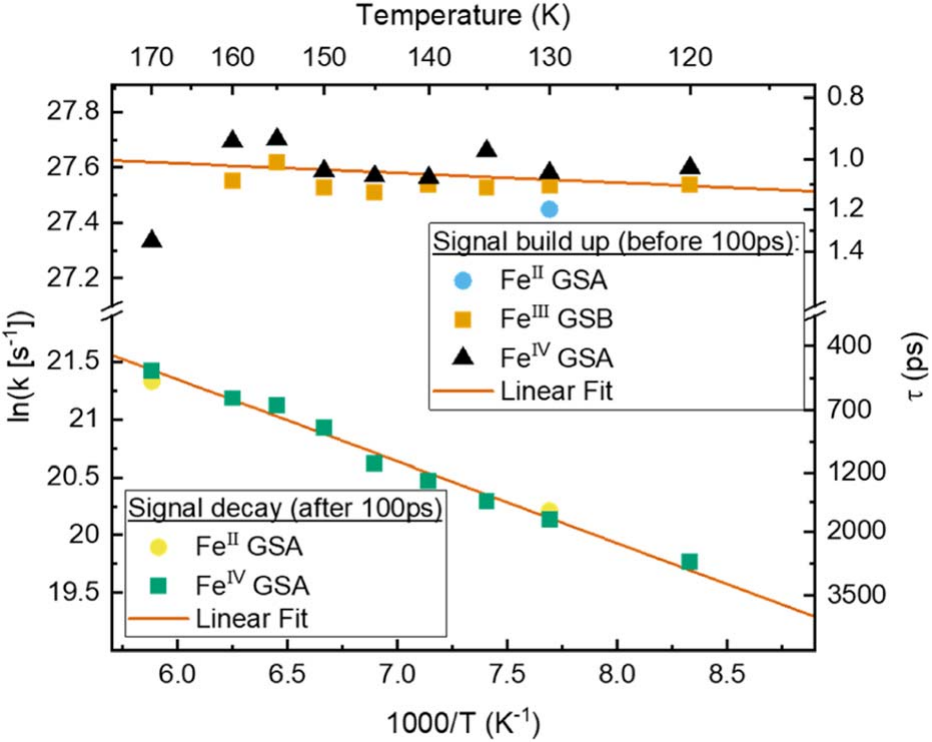
Arrhenius plot of signals associated with charge separation: build-up of Fe^II^ and Fe^IV^ GSA as well as Fe^III^ GSB, and associated with charge recombination: decay of Fe^II^ and Fe^IV^ GSA based on fitted components from TA of [Fe^III^(phtmeimb)_2_]PF_6_ in MeOH : EtOH. With linear fit in the selected temperature region.

To gain a further understanding of the underlying dynamics and the conversion between the different species all kinetics were fitted individually by the least sum of exponentials required to reproduce the data for each temperature. The fitting results are exemplified in Fig. 5b. All fit results are presented in Table S10.† The time-constants associated with CS are only observable below 170 K, *i.e.*, when Fe^II^- and Fe^IV^-species dominate the signal. The results can be separated in signal build up and decay with 100 ps being the inflection point between those two signals. Both, build up and decay are plotted in an Arrhenius-type plot in Fig. 6. The build-up of Fe^II^-species GSA (increase of positive signal at 350 nm), build-up of Fe^III^-species GSB (increase of negative signal at 450 nm), and build-up of Fe^IV^-species GSA (increase of positive signal at 710 nm) are indicative of CS. Similarly, the signal decay dynamics at 350 nm and 710 nm are associated with the decrease of Fe^II^- and Fe^IV^-species and thus the CR of molecules that underwent CS. The strikingly similar build-up of all three species, as well as the decay of Fe^II^ and Fe^IV^ species clearly proves that photochemical CS is the underlying process.

Interestingly, the rate of CS appears to be virtually temperature independent at ∼1 ps^−1^, while CR shows a slight dependence on temperature. However, both rates resemble a linear behaviour in the Arrhenius-type plot. From a linear fit we determine a pre-exponential of 1.2 × 10^12^ s^−1^ and activation energy of 24 cm^−1^ for CS, as well as frequency factor 1.3 × 10^11^ s^−1^ and activation energy of 490 cm^−1^ for CR. Extrapolating the charge recombination rate to 300 K yields a rate of ∼1.25 × 10^−2^ ps^−1^, which is in good agreement with ∼5 × 10^−2^ ps^−1^ previously reported for very high concentration at room temperature. The thermodynamic driving force of CR is roughly 2 times higher than that of CS (−1.4 eV *vs.* −0.7 eV), still CS proceeds at a much faster rate^30^ indicating that CR takes place in the inverted Marcus regime.

**Fig. 7:**
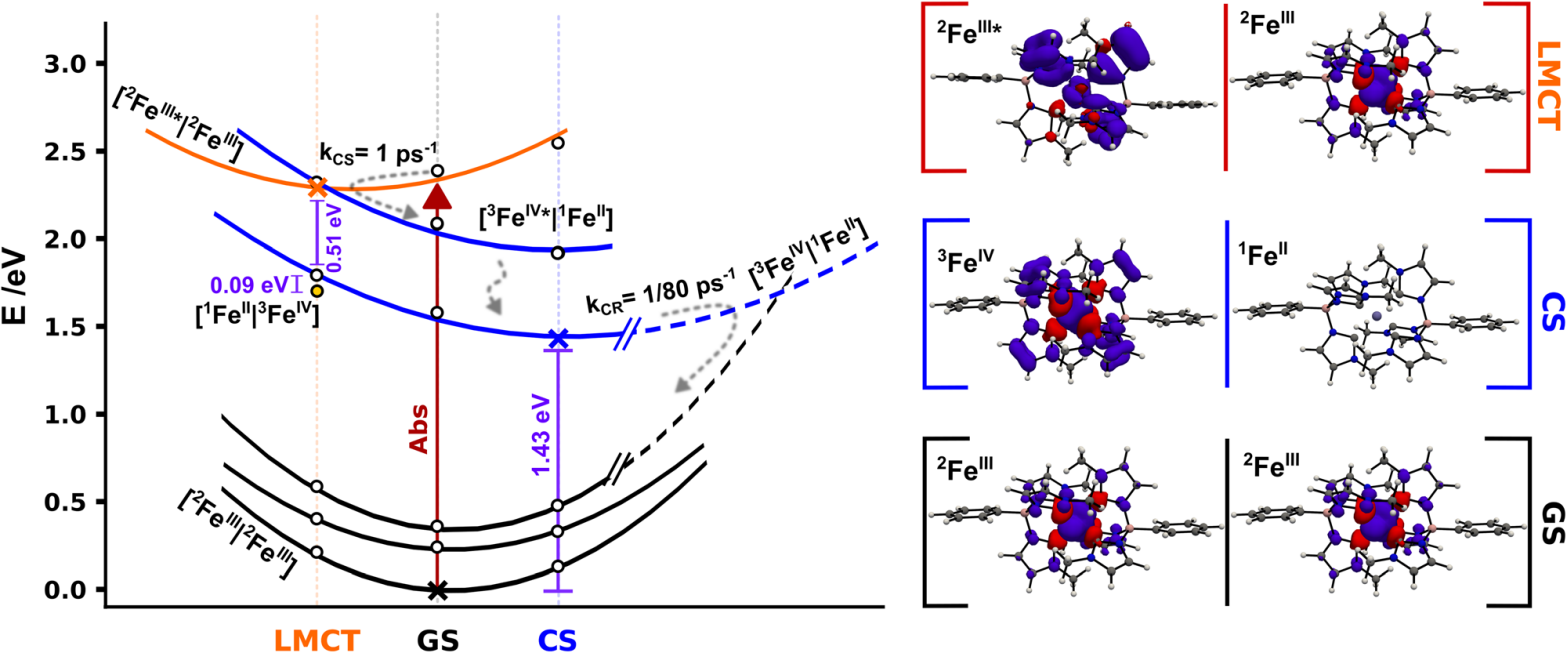
Schematic potential energy landscape for ^2^[Fe^III^(phtmeimb)_2_]^+^ pairs involved in photoinduced bimolecular charge separation and recombination based on DFT calculations. Cross markers correspond to the energy of the relaxed [^2^Fe^III^|^2^Fe^III^] (GS) and [^3^Fe^IV^|^1^Fe^II^] (CS) geometries and the [^2^Fe^III^*|^2^Fe^III^] (LMCT) minimum structure, white markers correspond to single point energy calculations for the respective relaxed structures. Yellow dot marks the energy of the [^1^Fe^II^|^3^Fe^IV^] state in the LMCT structure. Projected energy surfaces with visual guides for the various states represented in black for [^2^Fe^III^|^2^Fe^III^] states, in blue for [^3^Fe^IV^|^1^Fe^II^] and double degenerate [^3^Fe^IV^*|^1^Fe^II^] states, and in orange for the [^2^Fe^III^*|^2^Fe^III^] state. The right column shows spin densities for ^2^[Fe^III^(phtmeimb)_2_]^+^ GS, ^2^[Fe(phtmeimb)_2_]^+^ LMCT and relaxed ^3^[Fe^IV^(phtmeimb)_2_]^2+^ (full-size spin density pictures available in the ESI†).

To get further insight into the efficiency of CS and recombination, we investigate key aspects of the potential energy landscape through quantum-chemical calculations. The mechanism of CS in [Fe^III^(phtmeimb)_2_]^+^ after excitation was studied by performing DFT and TD-DFT calculations in a system described by two non-interacting iron complexes [Fe^N^|Fe^M^]. Computational outcomes are graphically represented in Fig. 7 (see ESI† for further details). First, we calculate the vertical electron excitation of one complex, *i.e.*, [^2^Fe^III^*|^2^Fe^III^] and subsequently consider structural relaxation towards the minimum energy geometry. The [Fe^III^(phtmeimb)_2_]^+ 2^LMCT state is a good photooxidant and photoreductant agent and thus two energetically degenerate CS states can be equally considered, [^1^Fe^II^|^3^Fe^IV^] and [^3^Fe^IV^|^1^Fe^II^], respectively. Since the ^1^Fe^II^ configuration is a closed-shell system, there are no close lying states expected. However, since ^3^Fe^IV^ is an open-shell system with three possible arrangements of the electrons in the three t_2g_ orbitals, a set of closely spaced excited states are expected. TD-DFT calculations predict two degenerate [^3^Fe^IV^*|^1^Fe^II^] and [^1^Fe^II^|^3^Fe^IV^*] excited states ∼0.5 eV above the corresponding deactivated states. Although the photoreduction of Fe^III^* requires ∼0.09 eV less energy than the photooxidation process, the [^3^Fe^IV^|^1^Fe^II^] and [^3^Fe^IV^*|^1^Fe^II^] states could have a more active role in the ^2^LMCT deactivation based on their closeness in energy.

The experimentally identified temperature independent CS implies a barrierless nature of the deactivation of the [^2^Fe^III^*|^2^Fe^III^] state if considered within the Marcus formalism. As the CS rate is high the process could occur before the complete thermalization of the excited system in a nonergodic fashion.^40^ This may be facilitated from an energy-gap perspective through the predicted presence of intermittently populated but short-lived [^3^Fe^IV^*|^1^Fe^II^] excited states (Fig. 7), although there is no empirical evidence of the former intermediate state. No shift of the Fe^IV^-species spectrum, that could indicate an Fe^IV^*-state, in TA was observed on the timescale investigated here. The reaction could be driven by fast intramolecular degrees of freedom or even by certain excitation-coupled vibrations prior to intramolecular vibrational energy redistribution.^41–43^ Previous reports on [Fe^IV^(phtmeimb)_2_]^2+^ revealed a fast decay of ∼1 ps of the excited states which suggests that the population of [^3^Fe^IV^*|^1^Fe^II^] undergoes rapid internal conversion to [^3^Fe^IV^|^1^Fe^II^]. The relaxed CS state was located wrapped in the GS with a calculated energy gap of 1.43 eV, in good agreement with the ∼1.4 eV reported in the literature.^30^ Furthermore, the calculations reveal that the one-electron oxidation and reduction of the respective Fe^III^ centres in the [^2^Fe^III^|^2^Fe^III^] couple results in minor structural distortions only. Based on the large energy separation of the CS state and the fact that CR occurs on a much slower time scale when complete thermalization in the excited system is expected we suggest that the CR proceeds as an activated process in the inverted Marcus region. Finally, we consider the overall efficiency of the photochemical CS in terms of the competition to other deactivation pathways. At 300 K the intramolecular deactivation exhibits a rate of 5 × 10^8^ s^−1^ (based on 2 ns luminescence lifetime). However, the virtually temperature independent intermolecular charge separation exhibits a rate of 10^12^ s^−1^. Considering that both the intra- and intermolecular deactivation pathways are largely governed by spin-allowed processes we note that the CS reaction outcompetes the intramolecular deactivation by almost four orders of magnitude, which is remarkable in a homogenous bimolecular system. The difference in rates corresponds to a quantum yield of 99.95% for charge separation of excited molecules given they have a suitable neighbouring molecule in the ground state to react with. Moreover, it is notable that this relative ratio of intramolecular *versus* intermolecular rates remains essentially unchanged over a wide temperature range owing to the fact that both the intra- and intermolecular deactivation processes show little temperature dependence. The thermodynamic driving force for bimolecular photochemical CS is −0.72 eV (−5800 cm^−1^) according to electrochemical measurements.^30^ The rather high driving force together with the very small activation energy of 3 meV (24 cm^−1^) renders the observed photoinduced disproportionation an especially efficient example of CS that easily outcompetes the intrinsic deactivation of [Fe^III^(phtmeimb)_2_]PF_6_ when close contact pairs of Fe(iii) complexes are present. The overall efficiency of the process is therefore limited by concentration and aggregation properties of the sample.

## Conclusions

In summary, [Fe^III^(phtmeimb)_2_]PF_6_ has been characterised by means of temperature dependent spectroscopy in various solvents in order to gain a thorough understanding of the excited state deactivation processes. In performing the first full Arrhenius analysis of a luminescent ^2^LMCT transition in a d^5^ transition metal complex, we reveal that the main intramolecular deactivation dynamics of this complex are only very weakly temperature dependent. In contrast to many iron and ruthenium d^6^ complexes where the decay *via* the ^3^MC state is limiting the lifetime, the activated decay *via* the ^4^MC state is not the dominant pathway. Instead, we observe that the inherent non-activated spin-allowed decay from ^2^LMCT to ^2^GS is now limiting the excited state lifetime and luminescence quantum yield of the complex. Whereas strong MC destabilization is important to avoid faster deactivation, it is evident by our study that further improvements in excited state lifetime will need to use other strategies mitigating the non-activated deactivation.

Our study further reveals the dynamics of intermolecular charge transfer, observed in aggregates induced by the low solubility of [Fe^III^(phtmeimb)_2_]PF_6_ and low temperature. The photoinduced disproportionation opens up an efficient pathway to quench the excited ^2^LMCT state that outcompetes the intramolecular deactivation by almost four orders of magnitude in the investigated temperature range. With both inter- and intra-molecular deactivation being spin-allowed, this is a surprising observation. The charge separation reaction is basically barrierless (activation energy of 3 meV) with a rather high thermodynamic driving force of −0.72 eV.^30^ Subsequent charge recombination takes place in the inverted Marcus regime, with an activation barrier of 60 meV. The barrier allows for ∼80 ps of expected corresponding lifetime of the charge-separated state at 300 K albeit a high thermodynamic driving force for recombination. Compared to previous reports of bimolecular reactions involving [Fe^III^(phtmeimb)_2_]PF_6_, this represents an order of magnitude slower charge recombination rate. Thus, by simply using the molecule itself as both acceptor and donor, it is possible to retain the fast charge separation but it significantly slows down the charge recombination. A benefit from this photoinduced disproportionation is the generation of two catalytically active species from only one excited molecule. It also demonstrates the versatility of this molecule, and suggests that the combination with other acceptor or donor systems with further improved energetics can result in even longer lived charge separated states.

Overall, our study highlights that the design strategy of destabilization of MC states and blocking unwanted intramolecular deactivation processes has been successfully implemented in [Fe^III^(phtmeimb)_2_]PF_6_. Simultaneously, we show that the main route for extending the excited state lifetime of the d^5^ complexes with the ^2^LMCT excited state lies in blocking unwanted spin-allowed direct deactivation. Additionally, the feasibility of efficient, ultrafast (∼1 ps) charge separation driven by the excited [Fe^III^(phtmeimb)_2_]PF_6_ complex highlights its true potential and reveals that 2 ns excited state lifetime truly is no impediment for photochemical applications using this Fe-based dye molecule.

## Data availability

The authors will provide data upon request.

## Author contributions

Nils W. Rosemann and Linnea Lindh performed and analysed the spectroscopy measurements and wrote major parts of the manuscript. Iria Bolaño Losada, performed quantum chemical calculations and provided analysis of these results to the manuscript. Simon Kaufhold, Om Prakash, Aleksandra Ilic, Jesper Schwarz and Kenneth Wärnmark synthesized the investigated metal complex. Pavel Chábera contributed to the transient absorption experiments as well as the analysis and discussion of the experimental results. Arkady Yartsev and Petter Persson conceived the project, participated in the analysis and discussions of the results, and wrote the manuscript.

## Conflicts of interest

There are no conflicts to declare.

## Supplementary Material

SC-014-D2SC05357H-s001
