# Supplementary material for: Competing dynamics of intramolecular deactivation and bimolecular charge transfer processes in luminescent Fe(iii) N-heterocyclic carbene complexes
Source: Chem Sci. 2023 Mar 7;14(13):3569–79. doi: 10.1039/d2sc05357h (PMC10056060; doi:10.1039/d2sc05357h)
Supplement: SC-014-D2SC05357H-s001 [file SC-014-D2SC05357H-s001.pdf]

Electronic Supplementary Material (ESI) for Chemical Science.
This journal is © The Royal Society of Chemistry 2023

Supplementary Information

Competing Dynamics of Intramolecular Deactivation and Bimolecular Charge Transfer Processes in Luminescent Fe(III) N-Heterocyclic Carbene Complexes

Nils W. Rosemann,[a,b] Linnea Lindh,[b,c] Iria Bolaño Losada,[c] Simon Kaufhold,[d] Om Prakash,[d] Aleksandra Ilic,[d] Jesper Schwarz,[d] Kenneth Wärnmark,[d] Pavel Chábera,[b] Arkady Yartsev,*[b] and Petter Persson*[c]

[a] N.W. Rosemann,
Light Technology Institute
Karlsruhe Institute of Technology
Engesserstraße 13, DE-76131 Karlsruhe, Germany

[b] N.W. Rosemann, L. Lindh, P. Chábera, A. Yartsev
Department of Chemical Physics
Lund University
Box 124, SE-22100 Lund, Sweden
E-mail: arkady.yartsev@chemphys.lu.se

[c] L. Lindh, I. Bolaño Losada, P. Persson
Department of Theoretical Chemistry
Lund University,
Box 124, SE-22100 Lund, Sweden

[d] S. Kaufhold, O. Prakash, A. Ilic, J. Schwarz, Wärnmark
Centre for Analysis and Synthesis
Department of Chemistry
Lund University, Box 124, SE-22100 Lund, Sweden

E-mail: petter.persson@teokem.lu.se

Supplementary Information

Sample Concentrations & Solvents 2

Steady State Absorption Spectroscopy 3

Steady State Emission Spectroscopy 6

Transient Absorption Spectroscopy 11

Time-Correlated Single Photon Counting 19

Arrhenius Analysis and Decomposition 20

Radiative and Non-Radiative Rates & Emission Quantum Yield 21

Quantum Chemical Calculations 25

References 35

Sample Concentrations & Solvents

For sample preparation $[Fe^{(III)}(phtmeimb)_2]PF_6$ powder and the respective solvent was mixed in standard glass vials under ambient conditions following procedures established in prior studies. [1] In case of butyronitrile, propionitrile and the methanol and ethanol mixture, the amount of solvent and solute were estimated to give a targeted concentration. For the mixtures with 1-propanol and 2-propanol, the added amount of solute was higher than the solubility limit. To ensure maximum concentration, the solutions were sonicated for ~20min, filtered using a 0.45µm syringe filter (PFTE) and then topped up with additional 10% of pure solvent to avoid spontaneous precipitation.

1-propanol (anhydrous, 99.7%), 2-propanol (anhydrous, ACS reagent, ≥99.5%), butyronitrile (≥99), and propionitrile (purum, ≥99.0% (GC)) were purchased from Sigma-Aldrich. Methanol (ACS EMSURE®, ≥99.9%) and ethanol (EMPLURA®, Supelco®, ≥99.5%) were purchased from VWR.

To estimate the concentration of $[Fe^{(III)}(phtmeimb)_2]^+$ in the various samples prepared for the measurements, we performed standard UV/Vis measurements on each sample directly after preparation of the sample. The absorbance at 502 nm is then used to calculate the concentration based on the extinction coefficient at that wavelength (2950 $M^{-1}cm^{-1}$). [1]

An overview of the concentrations in all solvents used for different experiments is given in Table S1.

Table S1. Sample concentrations of $[Fe^{(III)}(phtmeimb)_2]^+$ used for different experiments. The MeOH:EtOH solvent system has a volume ratio of 1:4.

Solvent	Method	Concentration [µM]
MeOH:EtOH	Linear Absorption	340
	Steady State Emission	380
	Transient Absorption	427
	TCSPC	170
Butyronitrile	Linear Absorption	210
	Steady State Emission	112
	Transient Absorption	370
	TCSPC	405
1-Propanol	Linear Absorption	362
	Transient Absorption	362
	TCSPC	290
Propionitrile	Linear Absorption	458
	Transient Absorption	458
2-Propanol	Linear Absorption	139
	Transient Absorption	139

In Table S2 we have collected relevant physical properties for all solvent systems used in the manuscript.

Table S2. Sample concentrations of $[Fe^{(III)}(phtmeimb)_2]^+$ used for different experiments. The MeOH:EtOH solvent system has a volume ratio of 1:4.

Solvent	Abbreviation	Freezing point [K]	Glass Transition [2–4] [K]	Polarity [5] $[E_T^N]$
Methanol	MeOH	175	103	0.762
Ethanol	EtOH	159	97	0.654
Methanol Ethanol (1:4 volume mix)	MeOH:EtOH	162 (interpolated)	98	0.676 (interpolated)
1-Propanol	1-PrOH	147	100	0.617
2-Propanol	2-PrOH	185	115	0.546
Propionitrile	EtCN	170	---	0.398
Butyronitrile	PrCN	161	100	0.364

Steady State Absorption Spectroscopy

Steady-state absorption measurements in the UV-VIS region were performed in a Perkin Elmer Lambda 1050 Spectrophotometer. An Oxford Instruments Optistat DN bath cryostat was put into the absorption spectrometer. The temperature of the sample could be controlled from 80 to 300 K, from utilizing N_2(l) in this cryostat. Absorbance of all prepared samples was measured in a standard quartz-glass cuvette of path length 1 mm (Hellma – High Performance Quartz Glass). For reference the same cuvette with pure solvent was used for each measurement.

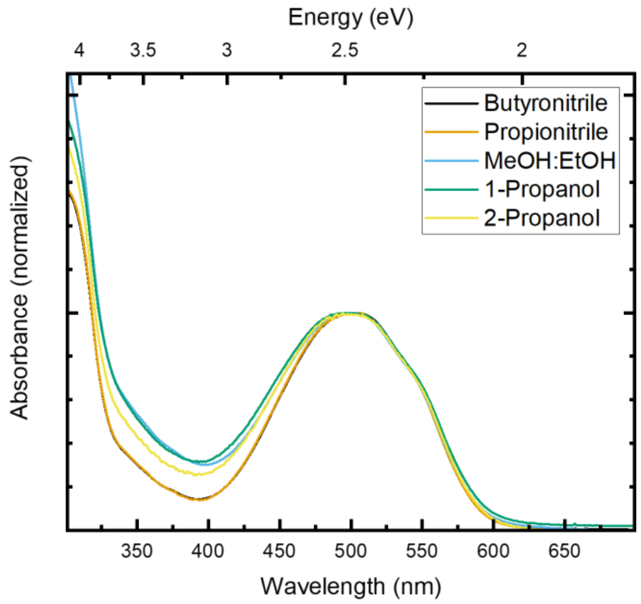

Figure S1. Steady state absorption at room temperature of $Fe(phtmeimb)_2]PF_6$ in all solvents used in this manuscript. The absorption spectra have been normalized for comparison.

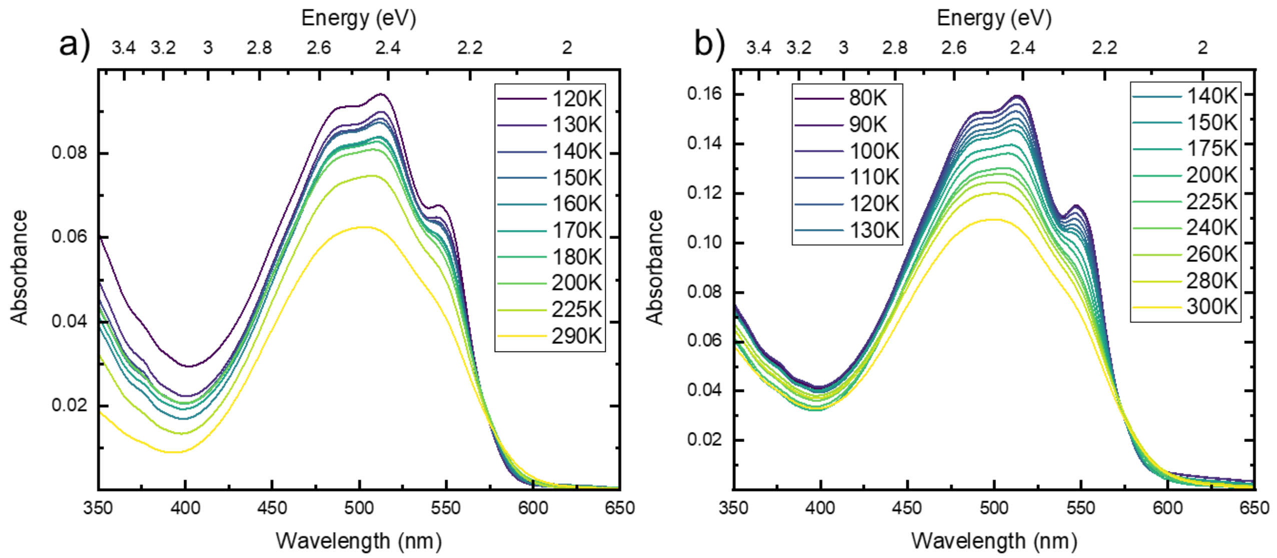

Figure S2. Steady state absorption spectra at all probed temperatures for Fe(phtmeimb)$_2$]PF_6 in a) butyronitrile and b) MeOH:EtOH (volume ratio 1:4).

For fitting of the absorption spectra, the spectra were first converted from wavelength scale to energy scale. Assuming that a vibronic progression is responsible for the observed multi-peak structure, the spectra are fitted by series of Gaussian peaks that share the same width and spacing to one another. With the general formula:

$$I(E) = \sum_{i=0}^{n} A_i \, e^{-\frac{(E-E_0+i\cdot\Delta E)^2}{2\omega^2}} \quad (1)$$

Where ΔE represents the spacing between the single peaks and ω the width. The results of these fits are given in Figure S3 for Butyronitrile as well as the MeOH:EtOH solvent mixture. In both cases the fits yield an overall agreement with the observed spectra. However, the distinct peaks of the experimental data are not well reproduced by the fits. Consequently, some of the restrains on the Gaussian peaks were lifted subsequently to improve the quality of the fit. Best results are obtained when using three Gaussian peaks that share the same width and spacing and two additional peaks with slightly different parameters. Resulting fits are shown exemplarily for the low temperature absorption spectra in Butyronitrile and MeOH:EtOH solvent mixture in Figure S4. For fitting of the whole temperature series, the parameters obtained at the lowest temperature were used as starting parameters for the next higher temperature and so forth. The fitting parameters obtained for the whole temperature series are summarized in Table S3 and Table S4.

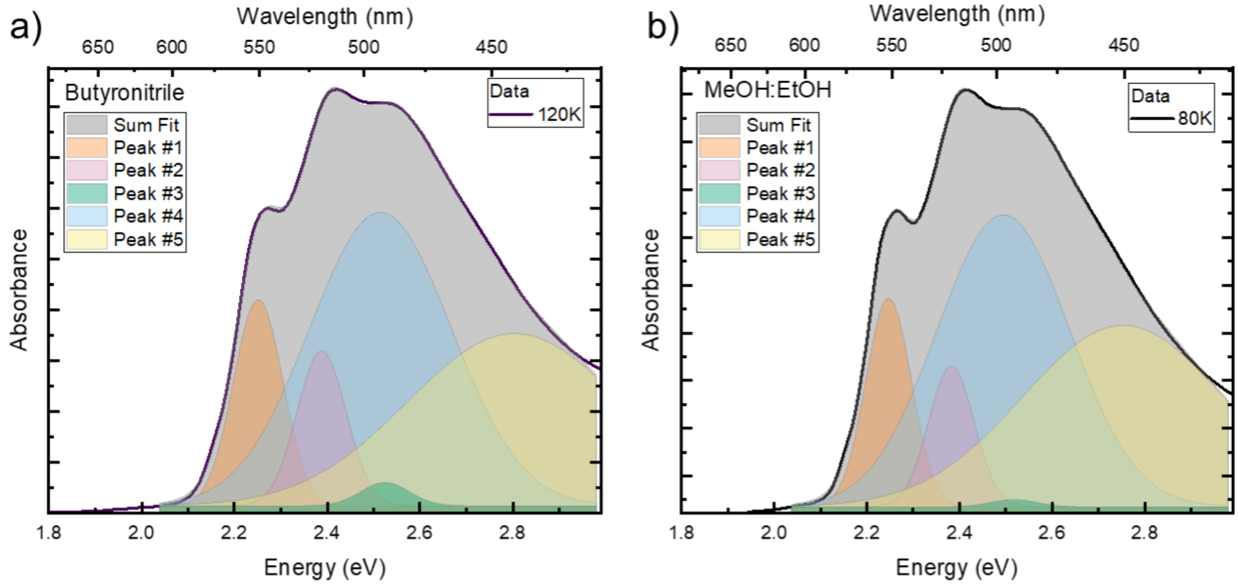

Figure S3. Steady state absorption spectrum at the lowest temperature measured of Fe(phtmeimb)$_2$]PF$_6$ in a) butyronitrile and b) MeOH:EtOH (volume ratio 1:4). Also included is the fit to the absorption spectrum using 5 Gaussian functions of shared width and spacing, modelling a vibrational progression. It is apparent that this model does not fit the measured data.

Figure S4. Steady state absorption spectrum at the lowest temperature measured of Fe(phtmeimb)$_2$]PF$_6$ in a) butyronitrile and b) MeOH:EtOH (volume ratio 1:4). Also included is the fit to the absorption spectrum using 5 Gaussian functions with less restrictions to width and spacing compared to fit results presented in Figure S3. It is apparent that this model provides a better fit

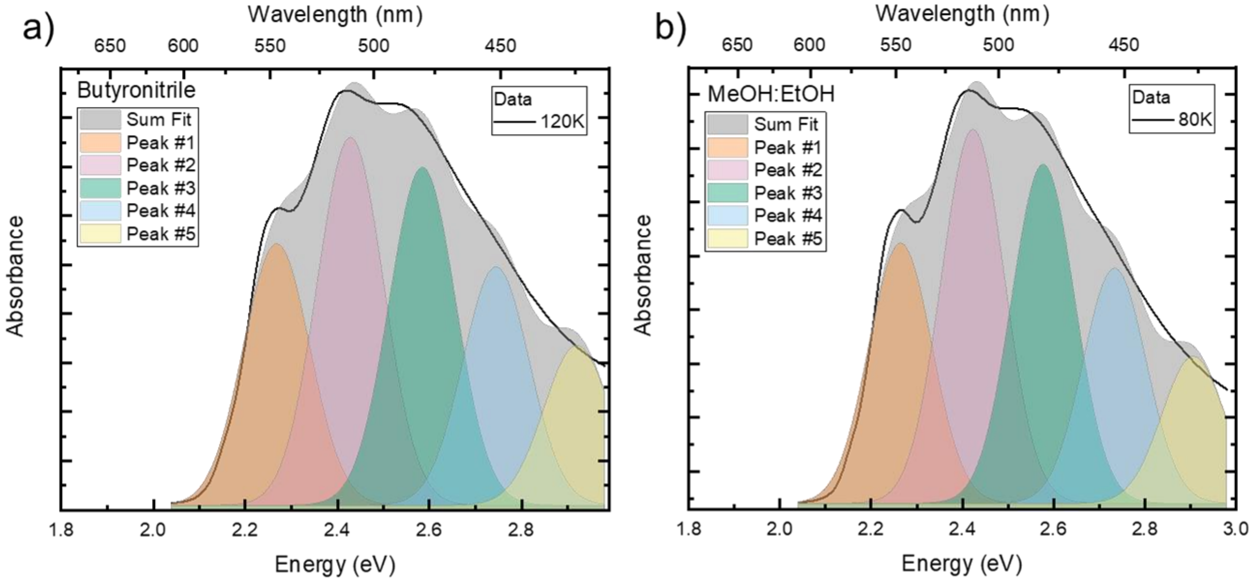

to the experimental data. The parameters obtained by the fits are listed in Table S3 and S4.

Table S3. Fitting parameters of the best obtained 5 Gaussian function fitting to the steady state absorption spectrum of Fe(phtmeimb)$_2$]PF$_6$ in MeOH:EtOH (volume ratio 1:4). The fit result are visualized in Figure S4.

Temperature (K)	Peaks #1-3						Peak #4			Peak #5		
	E_1 (eV)	Width (meV)	Spacing (meV)	A_1	A_2	A_3	E (eV)	Width (meV)	A	E (eV)	Width (meV)	A
80	2.25	49	135	0.077	0.052	0.003	2.75	217	0.067	2.50	143	0.107
100	2.25	50	136	0.075	0.052	0.003	2.76	219	0.066	2.50	145	0.107
120	2.25	51	137	0.066	0.044	0.004	2.77	235	0.063	2.50	156	0.105
140	2.25	52	138	0.061	0.042	0.005	2.78	241	0.060	2.50	161	0.102
175	2.25	60	140	0.061	0.048	0.010	2.80	231	0.053	2.53	160	0.093

200	2.26	64	141	0.060	0.049	0.011	2.79	226	0.049	2.53	162	0.090
225	2.26	67	143	0.056	0.048	0.012	2.82	233	0.044	2.54	168	0.086
260	2.26	73	145	0.052	0.047	0.014	2.87	242	0.040	5.56	173	0.082
300	2.26	78	147	0.046	0.042	0.011	2.85	231	0.036	2.57	168	0.070

Table S4. Fitting parameters of the best obtained 5 Gaussian function fitting to the steady state absorption spectrum of Fe(phtmeimb)$_2$]PF$_6$ in butyronitrile. The fit result is visualized in Figure S4 and Figure 1 (main manuscript).

Temperature (K)	Peaks #1-3						Peak #4			Peak #5		
	E_1 (eV)	Width (meV)	Spacing (meV)	A_1	A_2	A_3	E (eV)	Width (meV)	A	E (eV)	Width (meV)	A
120	2.25	53	137	0.046	0.034	0.005	2.52	153	0.065	2.81	230	0.038
140	2.25	56	137	0.043	0.035	0.007	2.52	153	0.059	2.78	220	0.033
160	2.25	59	138	0.041	0.033	0.008	2.53	156	0.055	2.78	217	0.029
180	2.25	62	140	0.038	0.033	0.009	2.54	163	0.056	2.82	229	0.027
200	2.26	65	141	0.037	0.033	0.009	2.55	165	0.054	2.83	231	0.026
225	2.26	68	142	0.034	0.030	0.008	2.55	163	0.048	2.79	217	0.023
290	2.26	78	143	0.027	0.026	0.007	2.55	158	0.036	2.76	201	0.020

Steady State Emission Spectroscopy

Prior to emission measurements the sample quality was checked by steady-state absorption and no significant differences from the known absorption spectra were found. Emission measurements were performed on a Horiba Fluorolog spectrofluorometer in the front face geometry, using a quartz cuvette with 1 cm or 1 mm optical path length (Hellma – QS-glass). To suppress stray light from sample excitation, long-pass filters were inserted for some measurements at the entrance slit of the detection monochromator. An Oxford Instruments Optistat DN bath cryostat was put into the emission spectrometer. The temperature of the sample could be controlled from 80 to 300 K, from utilizing N_2(l) in this cryostat. The recorded temperature dependent emission spectra over the whole accessible temperature range are plotted in Figure S5.

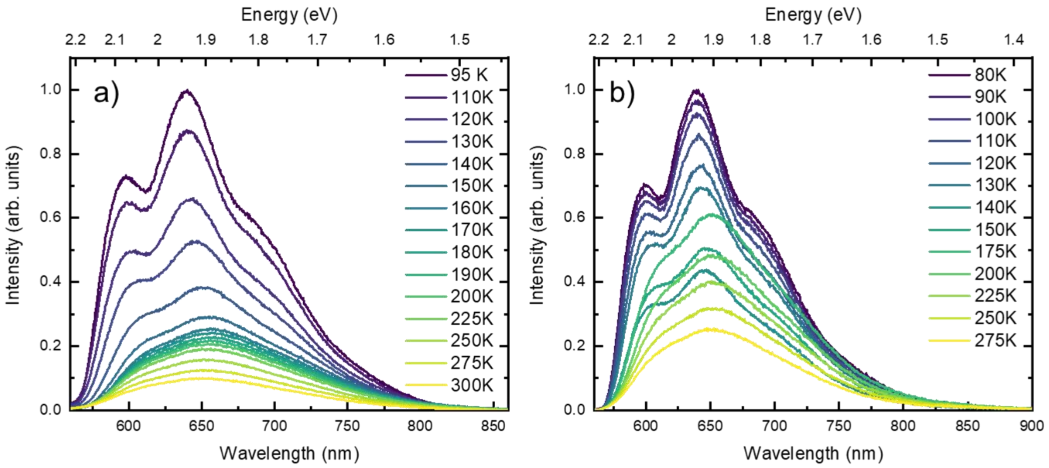

Figure S5. Steady state emission spectra at all probed temperatures for Fe(phtmeimb)$_2$]PF$_6$ in a) butyronitrile and b) MeOH:EtOH (volume ratio 1:4).

For fitting of the emission spectra, the spectra were first converted from wavelength scale to energy scale including the Jacobian conversion factor. [6] Subsequently the spectra are fitted by a series of Gaussian peaks. The best fitting results were obtained using 4 individual peaks with equal spacing and shared width. Resulting fits are shown exemplarily for the low temperature emission spectra in

Butyronitrile and MeOH:EtOH solvent mixture in Figure S6. For fitting of the whole temperature series, the parameters obtained at the lowest temperature were used as starting parameters for the next higher temperature and so forth. The obtained fitting parameters for the whole temperature series are summarized in Table S5 and Table S6.

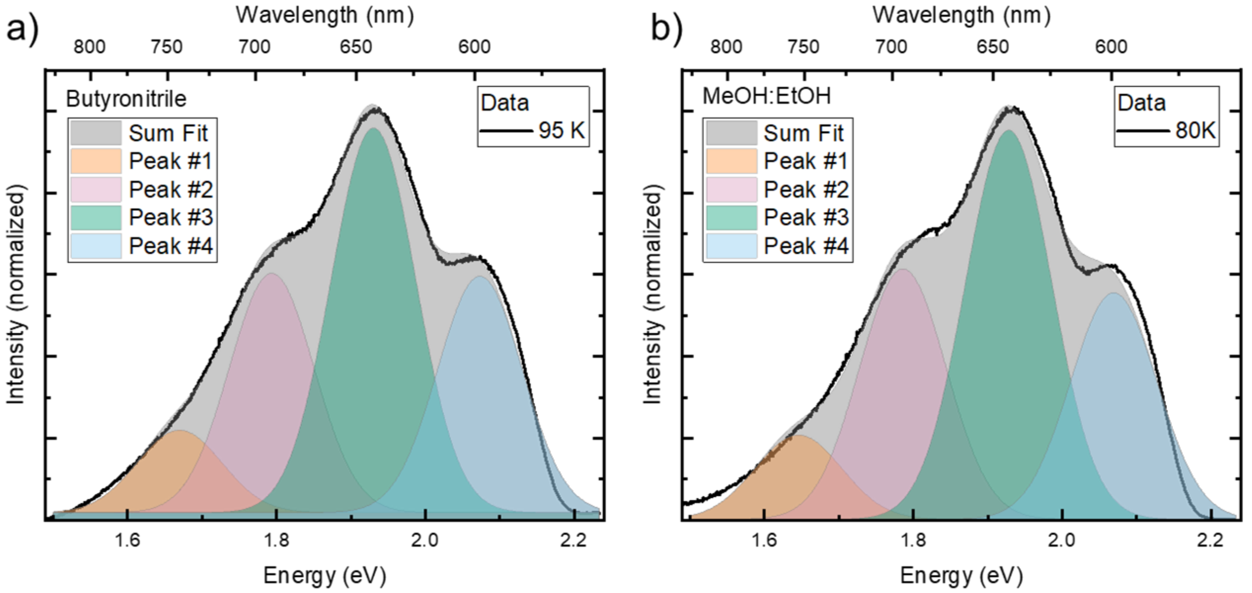

Figure S6. Low temperature steady state emission of Fe(phtmeimb)$_2$]PF_6 in a) butyronitrile and b) MeOH:EtOH (volume ratio 1:4) with fit by a series of Gaussian peaks. The individual Gaussian peaks are indicated by colored areas and their sum by the grey shaded area. The parameters obtained by the fits are listed in Table S5 and S6.

Table S5. Fitting parameters of the best obtained 4 Gaussian function fitting to the steady state emission spectrum of Fe(phtmeimb)$_2$]PF_6 in MeOH:EtOH (volume ratio 1:4). The fit result is visualised in Figure S6.

Temperature (K)	Shared width (meV)	Peak #1		Peak #2		Peak #3		Peak #4	
		E (eV)	A	E (eV)	A	E (eV)	A	E (eV)	A
80	58.2	2.07	11.77	1.93	20.20	1.79	13.00	1.65	4.379
90	57.7	2.07	14.44	1.93	19.58	1.79	12.45	1.65	4.138
100	58.0	2.07	11.03	1.93	11.03	1.79	11.88	1.65	3.986
110	58.6	2.06	10.41	1.92	17.20	1.79	10.91	1.65	3.615
120	59.5	2.06	9.477	1.92	15.29	1.78	9.712	1.64	3.202
130	60.2	2.05	8.866	1.91	13.95	1.78	8.818	1.64	2.867
140	61.4	2.05	5.666	1.91	8.673	1.77	5.463	1.64	1.736
150	63.3	2.04	6.629	1.90	10.01	1.76	6.358	1.63	1.976
175	67.0	2.02	7.898	1.89	11.80	1.75	7.702	1.61	2.348
200	69.1	2.02	6.139	1.88	9.157	1.74	5.981	1.61	1.786
225	70.1	2.02	5.009	1.88	7.472	1.74	4.934	1.60	1.425
250	71.6	2.02	3.955	1.88	5.895	1.74	3.851	1.60	1.109
275	72.5	2.02	3.118	1.88	4.634	1.74	3.015	1.60	0.845

Table S6. Fitting parameters of the best obtained 4 Gaussian function fitting to the steady state emission spectrum of Fe(phtmeimb)$_2$]PF$_6$ in butyronitrile. The fit result is visualized in Figure 1 (main manuscript) and Figure S6.

Temperature (K)	Shared width (meV)	Peak #1		Peak #2		Peak #3		Peak #4	
		E (eV)	A	E (eV)	A	E (eV)	A	E (eV)	A
95	56.4	2.08	23.33	1.94	32.44	1.80	17.74	1.67	5.713
110	57.7	2.07	20.75	1.93	29.38	1.79	15.66	1.66	4.915
120	62.0	2.07	11.05	1.92	14.42	1.79	8.149	1.66	2.616
130	62.8	2.06	9.987	1.92	13.04	1.79	7.445	1.66	2.359
140	63.4	2.06	89.00	1.92	11.62	1.78	6.670	1.65	2.084
150	63.8	2.05	76.74	1.91	10.04	1.78	5.760	1.65	1.784
160	66.5	2.05	57.65	1.91	7.547	1.77	4.345	1.64	1.317
180	71.0	2.02	73.29	1.88	9.925	1.75	6.069	1.62	1.755
200	71.6	2.02	65.38	1.89	8.922	1.76	5.404	1.63	1.628
225	73.3	2.03	59.48	1.89	8.171	1.76	4.855	1.63	1.449
250	74.7	2.03	48.29	1.89	6.716	1.76	3.988	1.63	1.139
275	76.7	2.03	37.54	1.89	5.233	1.76	3.102	1.63	0.8819
300	77.8	2.04	29.16	1.90	4.067	1.77	2.439	1.65	0.7433

To give an overview of the temperature dependent changes obtained by the fits, the individual peak positions relative to the lowest temperature position are plotted in Figure S7. Additionally, Figure S8 compares the temperature dependence of the fitted peak width in both solvent systems.

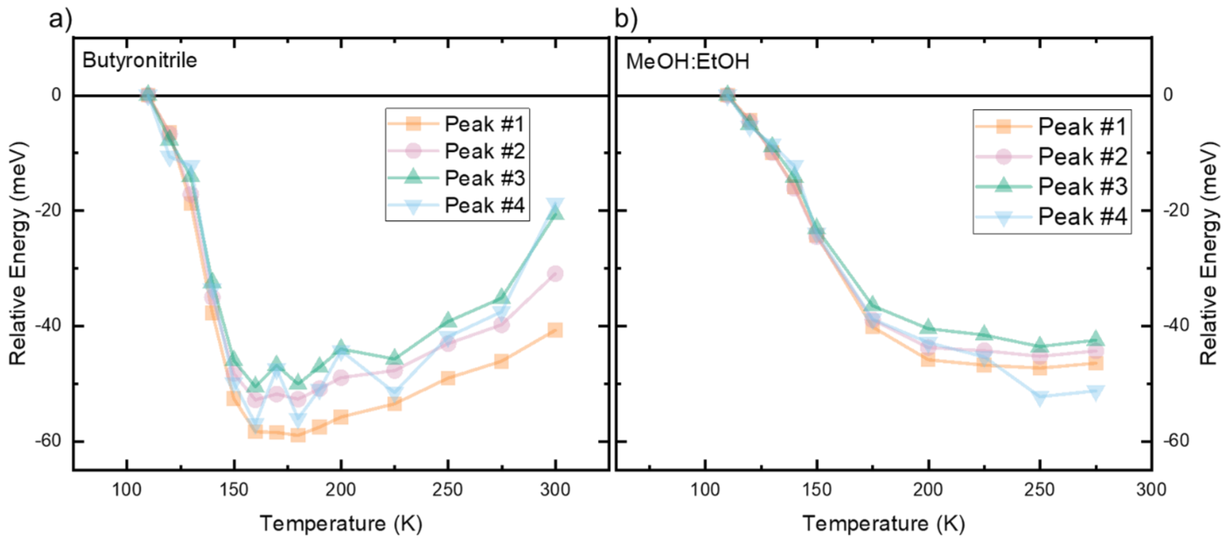

Figure S7. Temperature dependent steady-state emission characteristics of the relative emission peak shift of Fe(phtmeimb)$_2$]PF$_6$ in a) butyronitrile and b) MeOH:EtOH (volume ratio 1:4).

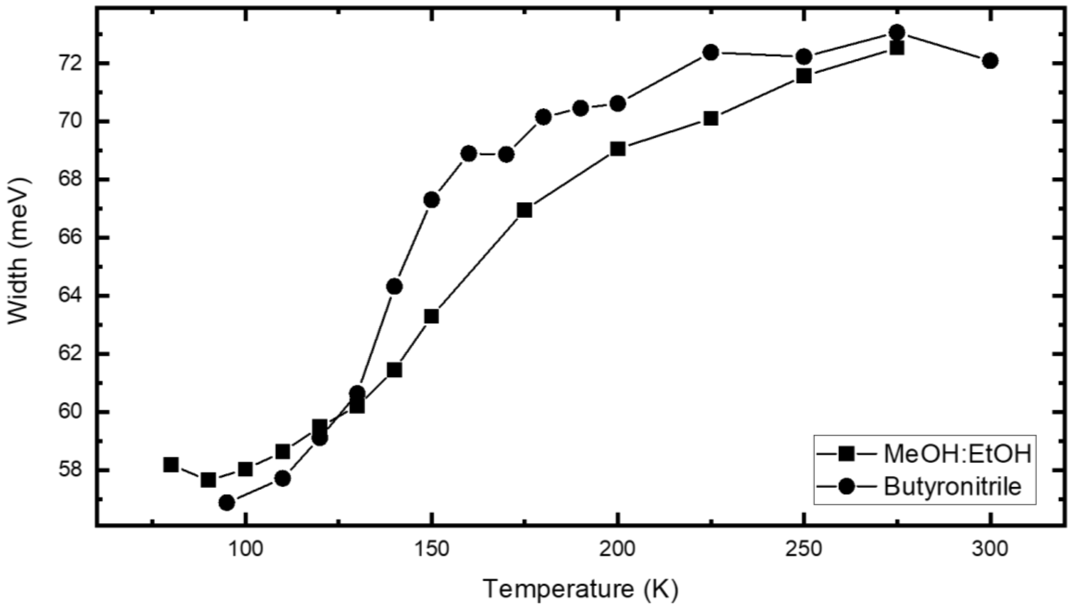

Figure S8. Temperature dependent steady-state emission characteristics of the peak width of Fe(phtmeimb)$_2$]PF$_6$ in both MeOH:EtOH (volume ratio 1:4) and butyronitrile.

In addition to fitting the spectra by a series of Gaussians, we also perform a Franck-Condon type analysis. [7] For this, the spectra are fitted by a series of Gaussian peaks that represent a vibronic progression with shared spacing and width. In contrast to the previous fit, where the individual amplitudes were used as fitting parameters, the amplitudes of the vibronic progression are scaled by the Huang-Rhys factor (S) that describes the coupling between electron and vibrational states and is related to the distortion between ground and excited state. [8,9] Best fits were obtained when using a model with two vibrational modes that is represented by:

$$I(E) = \sum_{\nu_1=0}^{N} \sum_{\nu_2=0}^{N} \left[\left(\frac{E_{00} - \nu_1 \hbar\omega_1 + \nu_2 \hbar\omega_2}{E_{00}} \right)^4 \left(\frac{S_1^{\nu_1}}{\nu_1!} \right) \left(\frac{S_2^{\nu_2}}{\nu_2!} \right) \times e^{-4ln2\left(\frac{E - E_{00} + \nu_1 \hbar\omega_1 + \nu_2 \hbar\omega_2}{\Delta\nu_{\frac{1}{2}}} \right)^2} \right] \quad 2$$

Here $\hbar\omega_1$ and $\hbar\omega_2$ give the energy of the vibronic modes that are involved. The factor $\Delta\nu_{\frac{1}{2}}$ gives the full width at half maximum of the observed Gaussian. The sum indices ν_1 and ν_2 define how many vibronic replicas are considered. In our case, a maximum of $N = 10$ was used to fit the spectra. The parameters S_1 and S_2 are the Huang-Rhys parameters describing the coupling to the respective vibrational modes. The energy E_{00} corresponds to the lowest energy transition between ground and excited state of the molecule.

Two exemplary fits of the lowest temperature emission spectra in butyronitrile and MeOH:EtOH solvent mixture are given in Figure S9. The overall shape of the observed emission spectra is reproduced well by the Franck-Condon analysis. However, compared to the fits by a simple series of

Gaussian peaks, the Franck-Condon analysis fails to adequately reproduce simultaneously both the high-energy and low-energy part of the emission spectrum.

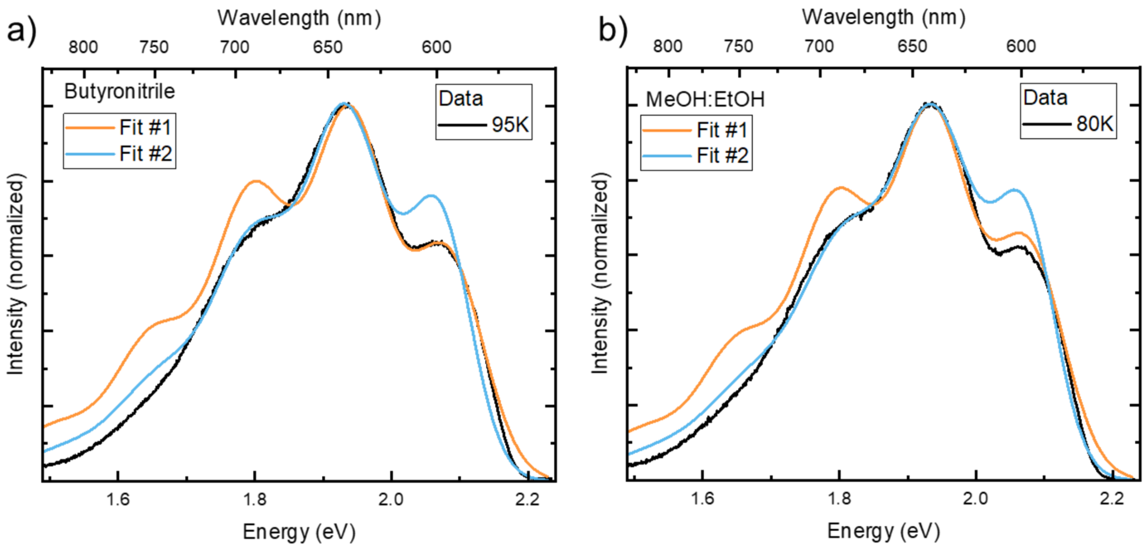

Figure S9. Franck-Condon analysis of the low temperature steady-state emission of Fe(phtmeimb)$_2$]PF_6 in both a) butyronitrile and b) MeOH:EtOH (volume ratio 1:4). For each solvent two fits were obtained. The first fit (orange line) is adapted to best fit the high-energy flank of the spectrum. The second fit (blue line) is adapted to best fit the intermediate region of the spectrum. Parameters of both fits are listed in Table S7 and S8, respectively.

Table S7. Fitting parameters of the Franck-Condon analysis of Fe(phtmeimb)$_2$]PF_6 in butyronitrile. The respective fit curves are visualised in Figure S9.

Fit	E_{00} (eV)	$\Delta\nu_{½}$ (meV)	$\hbar\omega_1$ (meV)	S_1	$\hbar\omega_2$ (meV)	S_2
#1	2.08	370	146	1.98	91	0.254
#2	2.07	356	146	1.35	89	0.65

Table S8. Fitting parameters of the Franck-Condon analysis of Fe(phtmeimb)$_2$]PF_6 in MeOH:EtOH (volume ratio 1:4). The respective fit curves are visualised in Figure S9.

Fit	E_{00} (eV)	$\Delta\nu_{½}$ (meV)	$\hbar\omega_1$ (meV)	S_1	$\hbar\omega_2$ (meV)	S_2
#1	2.08	122	144	1.9	91	0.25
#2	2.07	105	146	1.21	89	0.782

Transient Absorption Spectroscopy

Transient absorption (TA) spectroscopy was performed using an in-house built setup. Basis of this setup is a Spitfire Pro XP (Spectra Physics) laser amplifier system that produces ~80 fs pulses at a central wavelength of 796 nm at 1 kHz repetition rate. The amplifier output is divided into two parts that each pump collinear optical parametric amplifiers (TOPAS-C, Light Conversion). One of the TOPAS generates the pump beam (wavelength roughly set to the absorption maximum of each sample), while the other one generates a NIR beam (1350 nm) that is focused onto a 5 mm CaF_2 crystal to generate a supercontinuum probe beam. The delay between pump and probe beams is introduced by a computer-controlled delay stage (Aerotech) placed in the probe beam's path. After supercontinuum generation the probe pulses are split into two parts: the former being focused to ~100 µm spot size and overlapping with the pump pulse in the sample volume, and the latter serving as a reference. The sample is placed inside an Oxford Instruments Optistat DN bath cryostat and put into the spectrometer. The temperature of the sample could be controlled from 80 to 300 K, from utilizing N_2(l) in this cryostat. After passing the sample the probe beam is collimated again and relayed onto the entrance slit of a prism spectrograph. The reference beam is directly relayed on said spectrograph. Both beams are then dispersed onto a double photodiode array, each holding 512 elements (Pascher Instruments). The excitation power of the pulses was set to 1 mW at ~500 nm. Mutual polarization between pump and probe beams was set to the magic angle (54.7°) by placing a Berek compensator in the pump beam. Time-resolution of the setup after dispersion correction is estimated to be ≤150 fs.

The solution of $Fe(phtmeimb)_2]PF_6$ in different solvents (from Sigma-Aldrich Sure/Seal Bottle) was filled in 1 mm optical path length cuvettes (Hellma – Optical Special Glass). The measured samples were translated after each scan to avoid photodegradation. To check for stability of each sample steady-state absorption spectra were measured before and after TA experiments, and they were found to be the same. Before analysis the measured data were corrected for group velocity dispersion (GVD – "chirp").

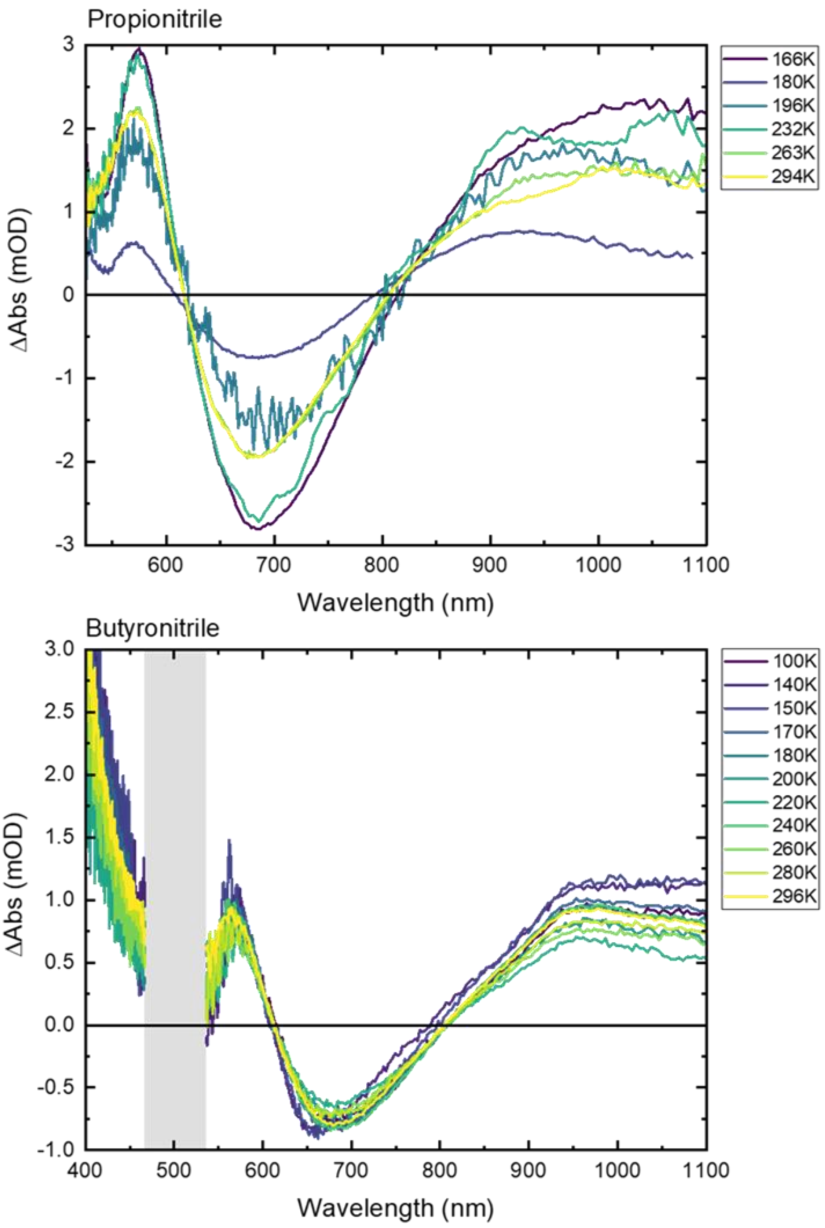

Figure S10. Transient absorption spectra of Fe(phtmeimb)$_2$]PF_6 in different high solubility solvents (nitriles) at all recorded temperatures. All spectra are recorded 100 ps after excitation at 500 nm. For clarity all spectra have been cut to remove excitation scatter, chirp (group velocity dispersion) and background corrected. Note that data is not normalized, but is represented as measured.

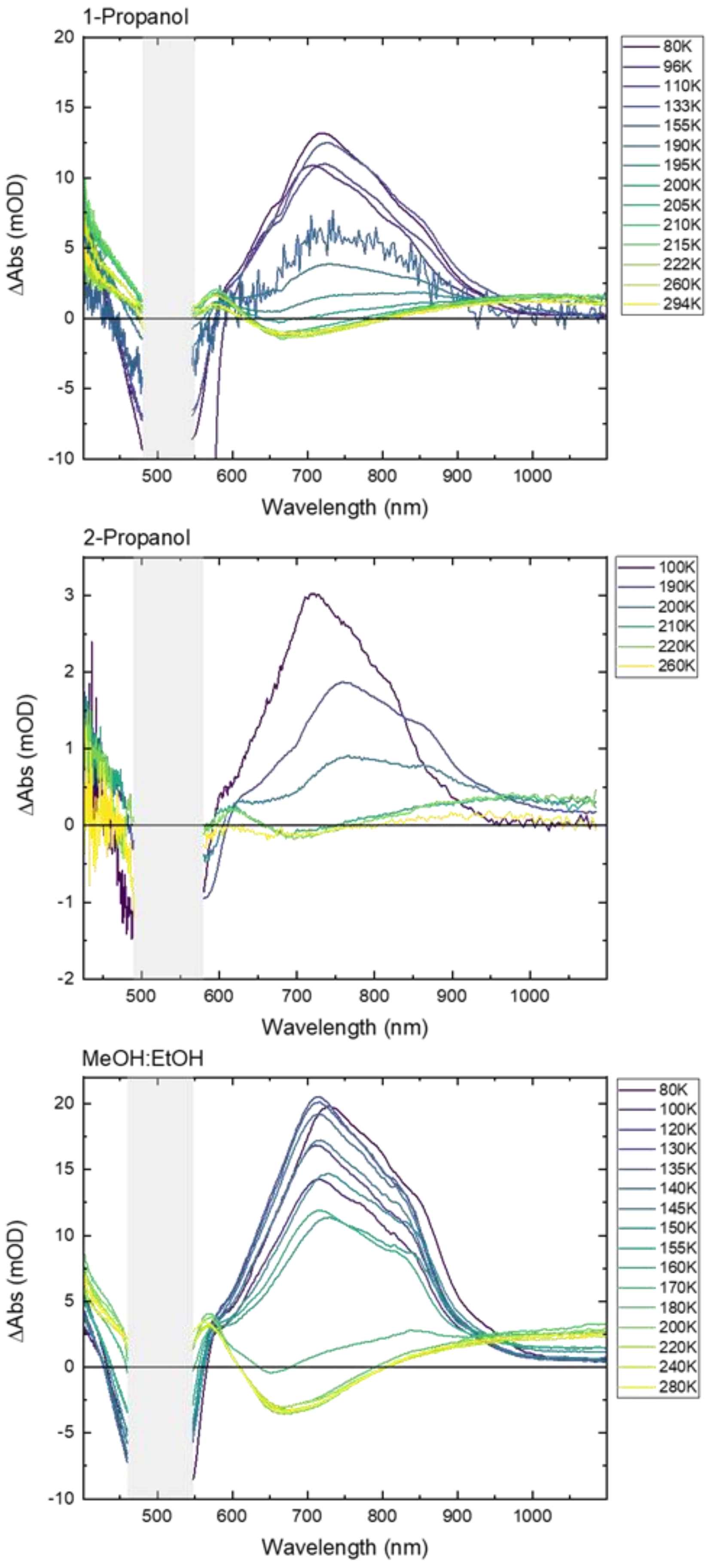

Figure S11. Transient absorption spectra of Fe(phtmeimb)$_2$]PF$_6$ in different low solubility solvents (alcohols) at all recorded temperatures. All spectra are recorded 100 ps after excitation at 500 nm. For clarity all spectra have been cut to remove excitation scatter, chirp (group velocity dispersion) and background corrected. Note that data is not normalized, but is represented as measured.

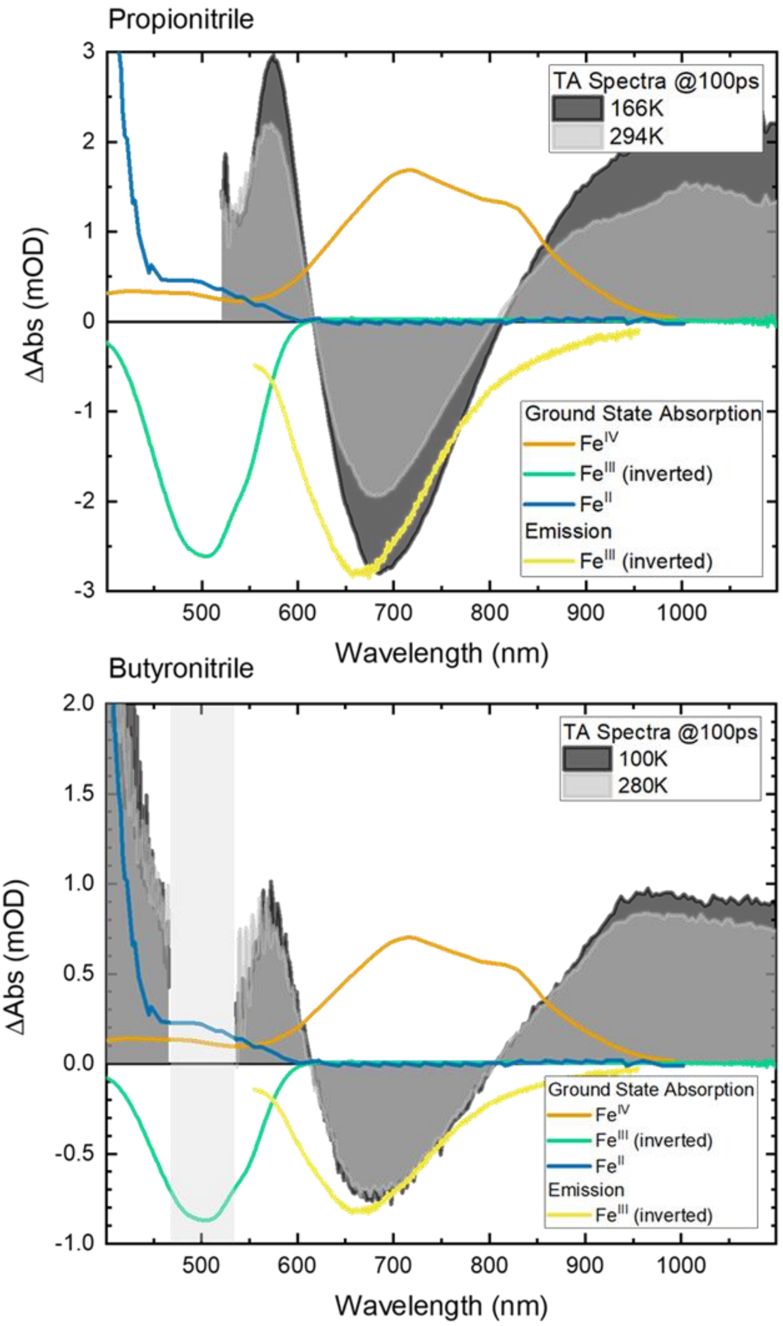

Figure S12. Transient absorption spectra of Fe(phtmeimb)$_2$]PF$_6$ compared in different solvents as well as at low temperature versus room temperature. All spectra are recorded 100 ps after excitation at 500 nm. For clarity all spectra have been cut to remove excitation scatter, chirp (group velocity dispersion) and background corrected. Note that data is not normalized, but is represented as measured. Added for comparison are also the steady state absorption spectra of [Fe^{IV}(phtmeimb)$_2$][PF$_6$]$_2$, [Fe^{III}(phtmeimb)$_2$]PF$_6$ (inverted) and Fe^{II}(phtmeimb)$_2$, together with the emission spectrum of [Fe^{III}(phtmeimb)$_2$]PF$_6$ (inverted).

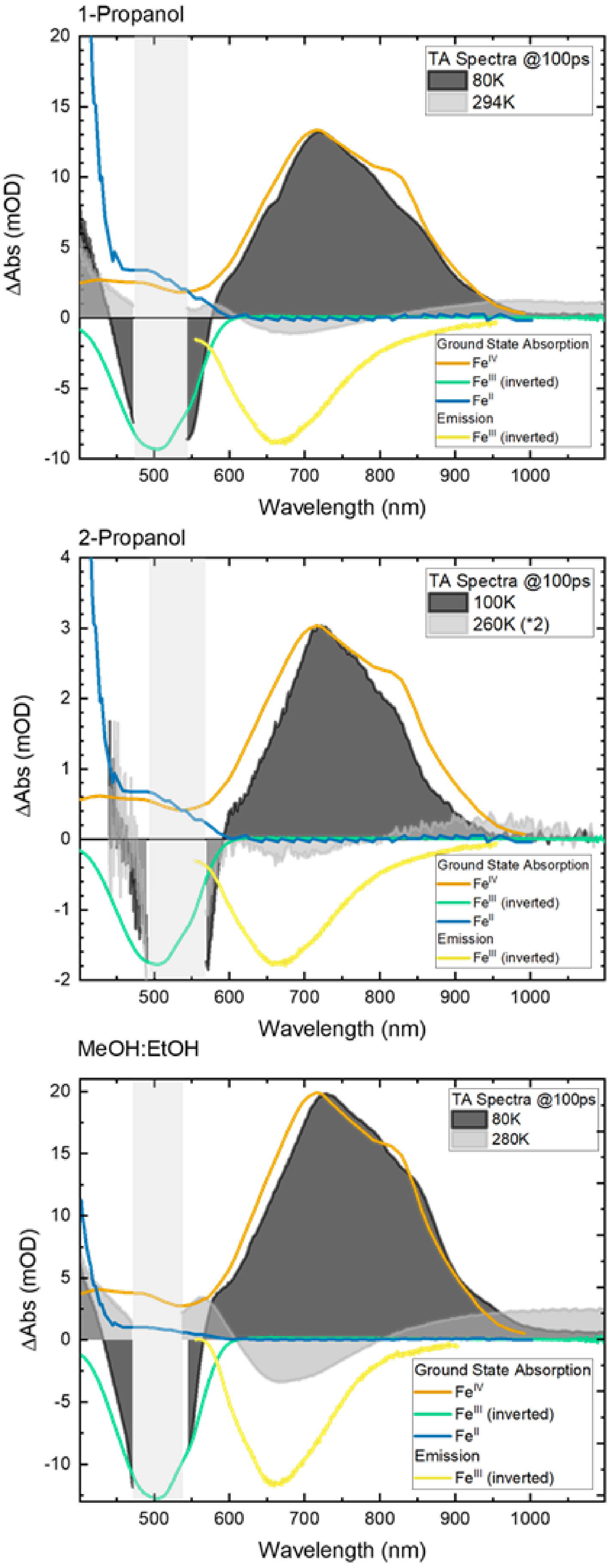

Figure S13. Transient absorption spectra of Fe(phtmeimb)$_2$]PF$_6$ compared in different solvents as well as at low temperature versus room temperature. All spectra are recorded 100 ps after excitation at 500 nm. For clarity all spectra have been cut to remove excitation scatter, chirp (group velocity dispersion) and background corrected. Note that data is not normalized, but is represented as measured. Added for comparison are also the steady state absorption spectra of [Fe^{IV}(phtmeimb)$_2$][PF$_6$]$_2$, [Fe^{III}(phtmeimb)$_2$]PF$_6$ (inverted) and Fe^{II}(phtmeimb)$_2$, together with the emission spectrum of [Fe^{III}(phtmeimb)$_2$]PF$_6$ (inverted).

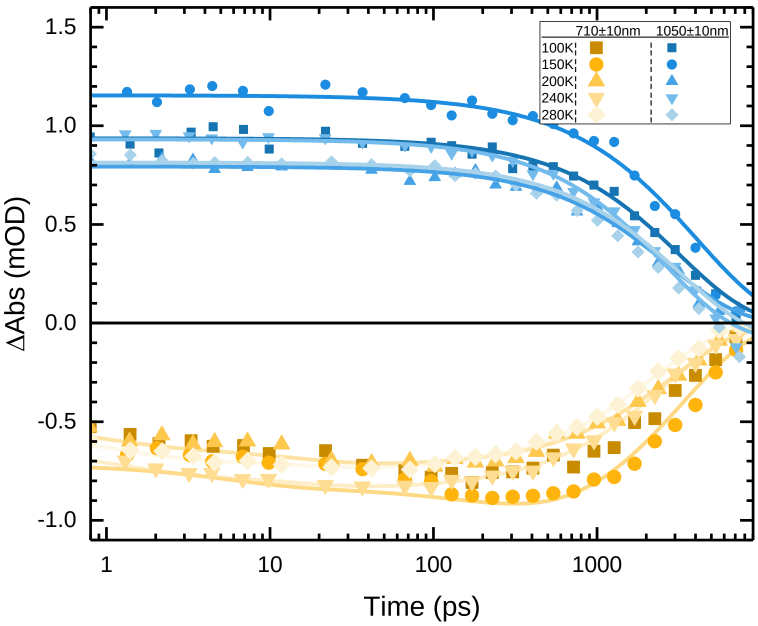

Figure S14. Transient absorption kinetics of Fe(phtmeimb)$_2$]PF$_6$ in PrCN at selected temperatures. Kinetics are recorded in the excited state absorption region (1050 nm) and in the stimulated emission region (710 nm). The ultrafast time evolution has here been omitted. Note that data is not normalized, but is represented as measured. Fits to the data are given as solid lines.

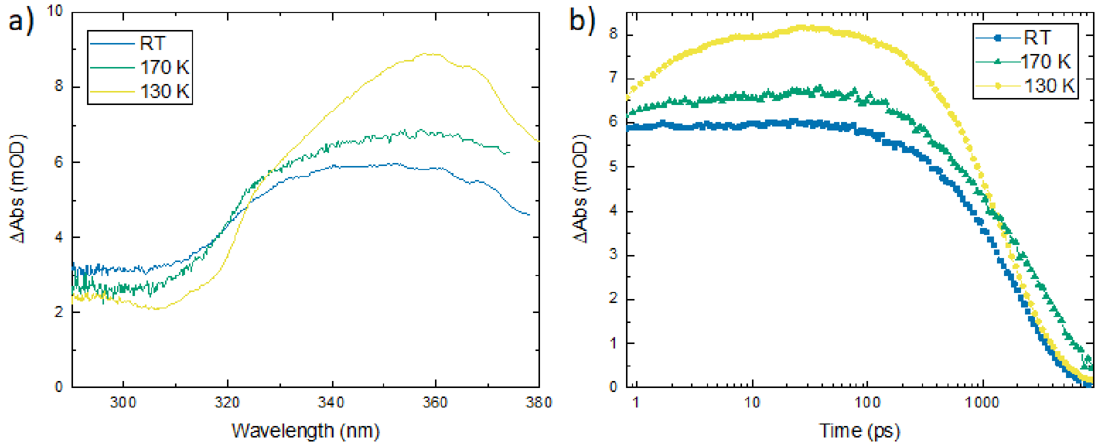

Figure S15. Transient absorption data of Fe(phtmeimb)$_2$]PF$_6$ in MeOH:EtOH (volume ratio 1:4) probed in the UV-wavelength range at selected temperatures. a) Spectra recorded 100 ps after excitation at 500 nm. For clarity all spectra have been chirp (group velocity dispersion) and background corrected. b) Kinetics recorded at 350 nm, the ultrafast time evolution has here been omitted. Note that data is not normalized, but is represented as measured.

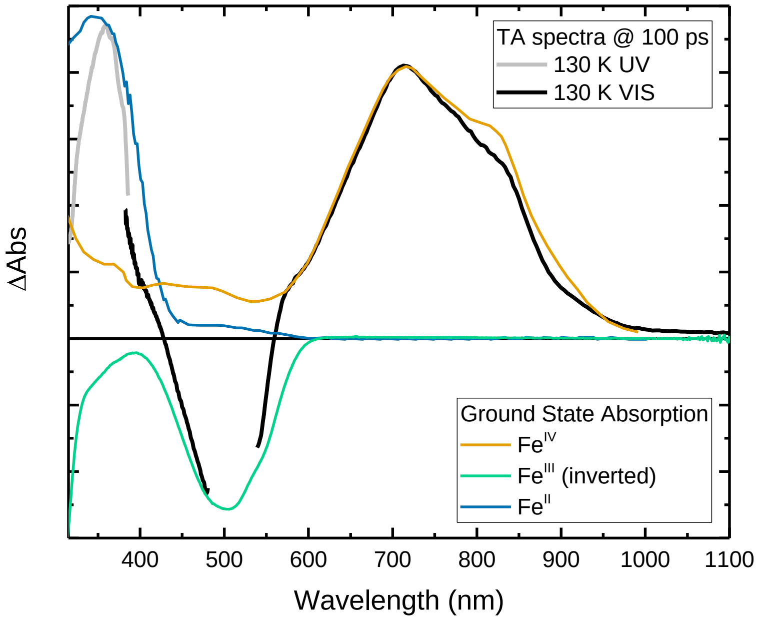

Figure S16. Transient absorption spectrum of [Fe(phtmeimb)$_2$]PF$_6$ in MeOH:EtOH (volume ratio 1:4) probed in the UV-wavelength range compared to a TA spectrum probed in the visible-NIR region. The two spectra are not plotted to scale, just for comparison. Spectra are recorded at 100 ps (+/-10 ps), at temperature 130 K. Added for comparison are also the steady state absorption spectra of [Fe^{IV}(phtmeimb)$_2$][PF$_6$]$_2$, [Fe^{III}(phtmeimb)$_2$]PF$_6$ (inverted) and [Fe^{II}(phtmeimb)$_2$].

Table S9. Fitted lifetimes at selected temperatures and wavelengths for [Fe(III)(phtmeimb)$_2$]$^+$ in butyronitrile. With arrows in parenthesis indicating time constants of signal intensities increasing (🡡) and decreasing (🡣), respectively.

Lifetimes	710 nm<br><br>[Fe(III)-SE]<br>(negative)	1050 nm<br><br>[Fe(III)-ESA]<br>(positive)
140 K	τ_1: 23ps (🡡)<br>τ_3: 780ps (🡡)<br>τ_3: 4600ps (🡣)	τ_1: 7200ps (🡣)
170 K	τ_1: 1.4ps (🡡)<br>τ_3: 30ps (🡡)<br>τ_3: 3600ps (🡣)	τ_1: 3300ps (🡣)
200 K	τ_1: 1ps (🡡)<br>τ_3: 15ps (🡡)<br>τ_3: 3000ps (🡣)	τ1: 2600ps (🡣)
260 K	τ_1: 1ps (🡡)<br>τ_3: 24ps (🡡)<br>τ_3: 2100ps (🡣)	τ_1: 2700ps (🡣)
280 K	τ_1: 1ps (🡡)<br>τ_3: 15ps (🡡)<br>τ_3: 2200ps (🡣)	τ_1: 2500ps (🡣)
296 K	τ_1: 1ps (🡡)<br>τ_3: 15ps (🡡)<br>τ_3: 1700ps (🡣)	τ_1: 2100ps (🡣)

Table S10. Fitted lifetimes at selected temperatures and wavelengths for [Fe(III)(phtmeimb)$_2$]$^+$ in MeOH:EtOH (volume ratio 1:4). With arrows in parenthesis indicating time constants of signal intensities increasing (↑) and decreasing (↓), respectively.

Lifetimes [a]	350 nm<br><br>[Fe(II)-GSA]<br>(positive)	450 nm<br><br>[Fe(III)-GSB]<br>(negative)	450 nm<br><br>[Fe(III)-ESA]<br>(positive)	700 nm<br><br>[Fe(IV)-GSA]<br>(positive)	700 nm<br><br>[Fe(III)-SE]<br>(negative)	1050 nm<br><br>[Fe(III)-ESA]<br>(positive)
80 K		τ_1: 1.6ps (↑)<br>τ_2: 40ps (↑)<br>τ_3: 860ps (↓)		τ_1: 1ps (↑)<br>τ_2: 9ps (↑)<br>τ_3: 500ps (↓)<br>τ_4: 4700ps (↓)		τ_1: 1.8ps (↓)<br>τ_2: 4900ps (↓)
130 K	τ_1: 1.2ps (↑)<br>τ_2: 20ps (↑)<br>τ_3: 1700ps (↓)	τ_1: 1.1ps (↑)<br>τ_2: 10ps (↑)<br>τ_3: 120ps (↓)<br>τ_4: 1800ps (↓)		τ_1: 1ps (↑)<br>τ_2: 12ps (↑)<br>τ_3: 1800ps (↓)		τ_1: 1.8ps (↓)<br>τ_2: 3900ps (↓)
170 K	τ_1: 15ps (↑)[a]<br>τ_2: 550ps (↓)<br>τ_3: 4000ps (↓)		τ_1: 2700ps (↓)<br>τ_2: 450ps (↓)	τ_1: 0.3ps (↑)<br>τ_2: 1.3ps (↑)<br>τ_3: 500ps (↓)	τ_1: 3100ps (↓)	τ_1: 0.7ps (↓)<br>τ_2: 3600ps (↓)
200 K			τ_1: 3000ps (↓)		τ_1: 0.3ps (↑)<br>τ_2: 1.3ps (↑)<br>τ_3: 140ps (↑)<br>τ_4: 3200ps (↓)	τ_1: 3100ps (↓)
280 K[a]	τ_1: 2000ps (↓)		τ_1: 2100ps (↓)		τ_1: 0.7ps (↑)<br>τ_2: 11ps (↑)<br>τ_3: 2200ps (↓)	τ_1: 2100ps (↓)

a) For the wavelength 350 nm fitted at room temperature.

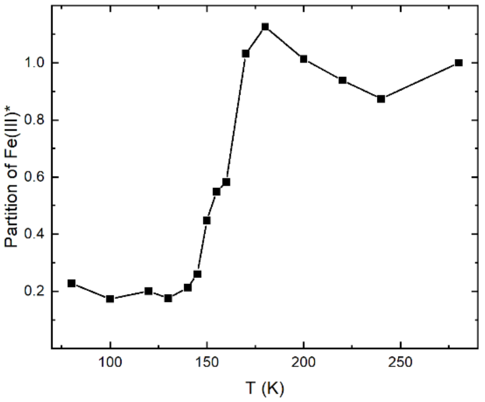

Figure S17. Estimation of the partition [Fe(III)(phtmeimb)$_2$]$^+$ complexes that are in the excited state at various temperatures in MeOH:EtOH (volume ratio 1:4) 100 ps after excitation. The complexes not in the excited state are expected to have undergone photoinduced disproportionation.

Time-Correlated Single Photon Counting

Time correlated single photon counting (TCSPC) was measured using a PicoQuant PicoHarp 300 system, with a Sepia II diode laser. The samples were mounted in an Oxford Instruments Optistat DN bath cryostat that was placed in the TCSPC setup. The temperature of the sample could be controlled from 80 to 300 K, from utilizing N_2(l) in this cryostat. For excitation, a 438 nm pulsed laser diode was focused onto the sample cuvette. The laser was triggered externally at 2.5 MHz, with pulse duration of approximately 200 ps. Detection was performed in right-angle geometry. For this, the sample is tilted 45° towards the excitation and in 45° to the surface normal a lens is imaging the sample onto an avalanche photodiode (SPAD, Micro Photon Device). The response time of the photodiode was <50 ps. To block stray light of the laser from entering the detection system and partially select the detection wavelength, a combination of 450 nm (Schott GG450) and 550 nm (Schott OG550) color glass long-pass filters was used.

The resulting kinetics for three different solvents at all investigated temperatures are shown in Figure S18. The normalized kinetics were fitted by a sum of exponential decays of the general formula:

$$I(t) = y_0 + A_1 e^{-\frac{t}{\tau_1}} + A_2 e^{-\frac{t}{\tau_2}} \quad 3$$

Below the freezing point of the solvent the second exponential decay had to be introduced to accurately reproduce the measured data. The resulting time constants and amplitudes at all measured temperatures and for the three solvents are summarized in Figure S19.

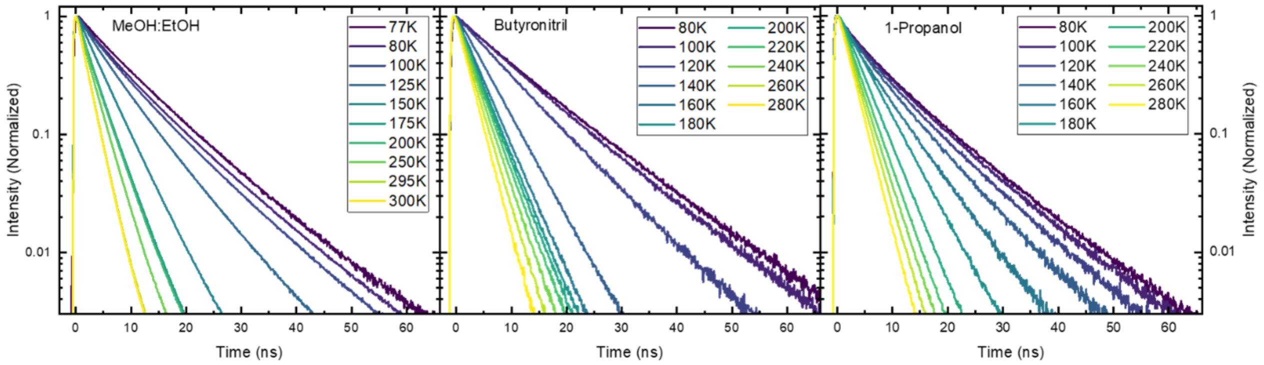

Figure S18. TCSPC kinetics at all probed temperatures for Fe(phtmeimb)$_2$]PF_6 in the three solvent systems MeOH:EtOH (volume ratio 1:4), butyronitrile and 1-propanol.

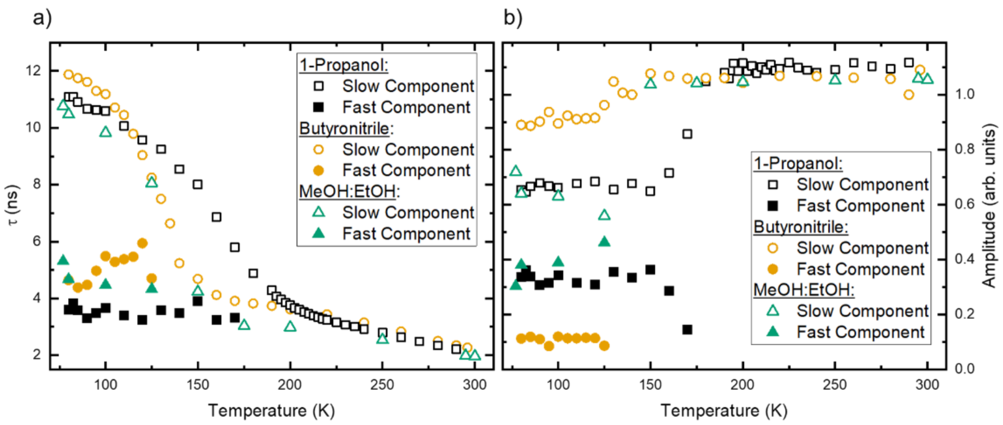

Figure S19. Fit result for TCSPC data for Fe(phtmeimb)$_2$]PF$_6$ in the three solvent systems 1-propanol, butyronitrile and MeOH:EtOH (volume ratio 1:4). Time constants (a) and amplitudes (b) determined by double exponential fit.

Arrhenius Analysis and Decomposition

The time resolved photoluminescence data obtained by TCSPC has been analyzed following a standard Arrhenius-type model: [10]

$$\frac{1}{\tau_{obs}} = k_0 + A_1 e^{-\frac{\Delta E_1}{RT}} + A_2 e^{-\frac{\Delta E_2}{RT}} + \frac{M}{1+e^{C(1/T-1/T_m)}} \quad 4$$

The model includes two activated terms with scaling factor A$_1$ and A$_2$, and one term that accounts for the phase-transition of the solvent (M-term). Based on the measured luminescence lifetime (τ_{obs}) and the fit results, we decompose Equation 4 into the individual summands. The results for [Fe^{III}(phtmeimb)$_2$]PF$_6$ in MeOH:EtOH and 1-propanol are plotted together with the respective data in Figure S20. For comparison we perform the same analysis on well established ruthenium complexes. The results for [Ru^{II}(dqp)$_2$][PF$_6$]$_2$ (with dpq being 2,6-di(quinoline-8-yl)pyridine) [7] and [Ru^{II}(pymbpy)$_2$][PF$_6$]$_2$ (with pymbpy being 6-(2-picolyl)-2,2'-bypridine) [11] in acetonitrile are plotted in Figure S21.

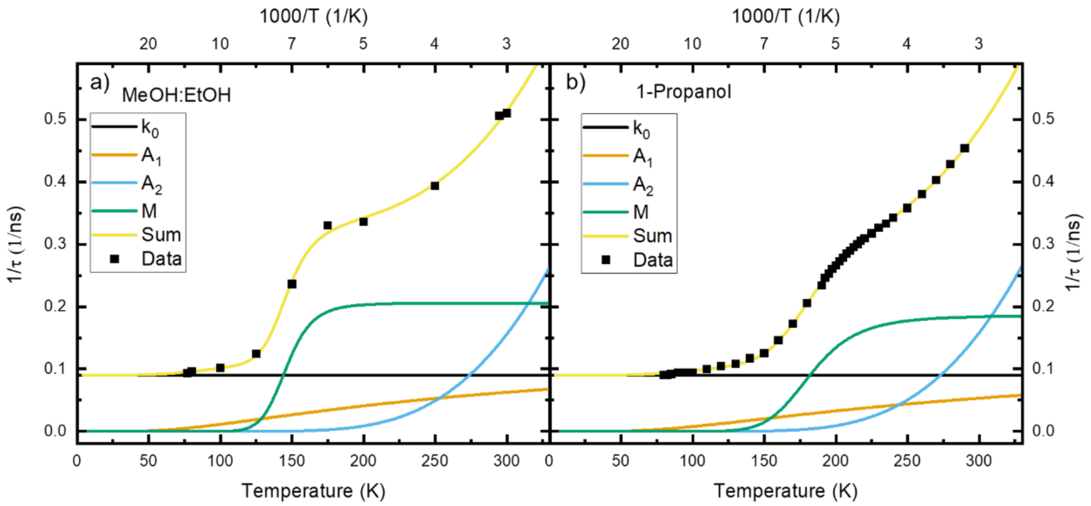

Figure S20. Arrhenius fit and decomposition of the individual components of $[Fe^{III}(phtmeimb)_2]PF_6$ in a) MeOH:EtOH mixture (volume ratio 1:4) and b) 1-propanol.

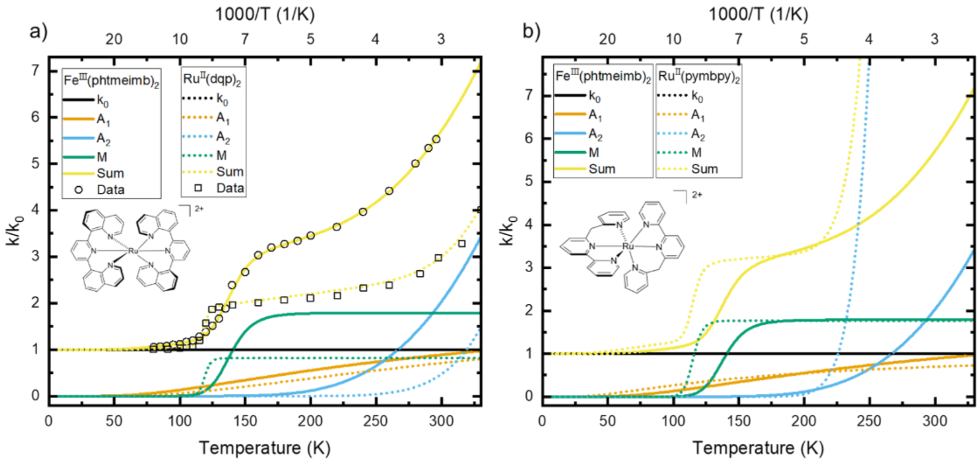

Figure S21. Arrhenius fit and decomposition of the individual components of $[Fe^{III}(phtmeimb)_2]PF_6$ in butyronitrile compared to other transition metal complexes, a) $[Ru^{II}(dqp)_2][PF_6]_2$ and b) $[Ru^{II}(pymbpy)_2][PF_6]_2$. [11,12]

Radiative and Non-Radiative Rates & Emission Quantum Yield

Based on the integrated extinction coefficient we estimate the radiative recombination rate (k_r) following the Strickler-Berg formalism:

$$k_r = 2.88 \cdot 10^{-9} \cdot \nu_{max}^2 \cdot n^2 \int \varepsilon(\nu) d\nu \qquad 5$$

With ν_{max} being the wavenumber of the absorption maximum, the refractive index of the solvent (n) and ε the extinction coefficient plotted versus wavenumber. [13] In combination with the measured luminescence lifetime (τ_{TCSPC}) we can then estimate the non-radiative recombination rate (k_{nr}) according to:

$$\frac{1}{\tau_{TCSPC}} = k_r + k_{nr} \qquad 6$$

Using the combination of radiative and non-radiative rate we estimate the emission quantum yield (η) by:

$$\eta = \frac{k_r}{k_r + k_{nr}} \quad (7)$$

The extinction coefficients are integrated from the absorption spectra in Figure S2 over the spectral region corresponding to the LMCT transition (400 nm – 600 nm). The resulting values for two different solvent systems are given in Figure S22. The increase in extinction coefficient appears to be independent from the solvent system. Below 100 K the extinction coefficient is no longer increasing but leveling off.

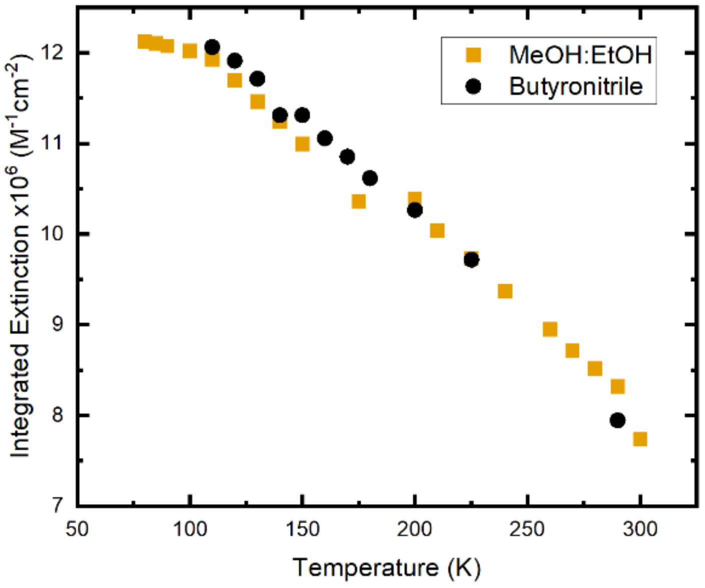

Figure S22. Temperature dependent extinction coefficient of $[Fe^{III}(phtmeimb)_2]PF_6$ in MeOH:EtOH (orange squares) and butyronitrile (black circles) integrated over the LMCT region of the absorption spectrum.

The calculated temperature dependent radiative and non-radiative recombination rates are presented in Figure S23 whereas the lifetimes are taken from Figure S19. For both solvents the radiative rates are found to be very similar. Additionally, the radiative rate increases only slightly from $1.5 \cdot 10^7$ s^{-1} at RT to $2.5 \cdot 10^7$ s^{-1} at 80 K. In the high- and low-temperature limit, the non-radiative rates are also similar for the two solvent systems. However, the non-radiative rate decreases by a factor of 10 from ~$5 \cdot 10^8$ s^{-1} at RT to $0.5 \cdot 10^8$ s^{-1} at 80 K. In the intermediate temperature range the two solvents show slightly different changes of the non-radiative decay rates. This is attributed to the differences in freezing point and glass transition temperature of the two solvents.

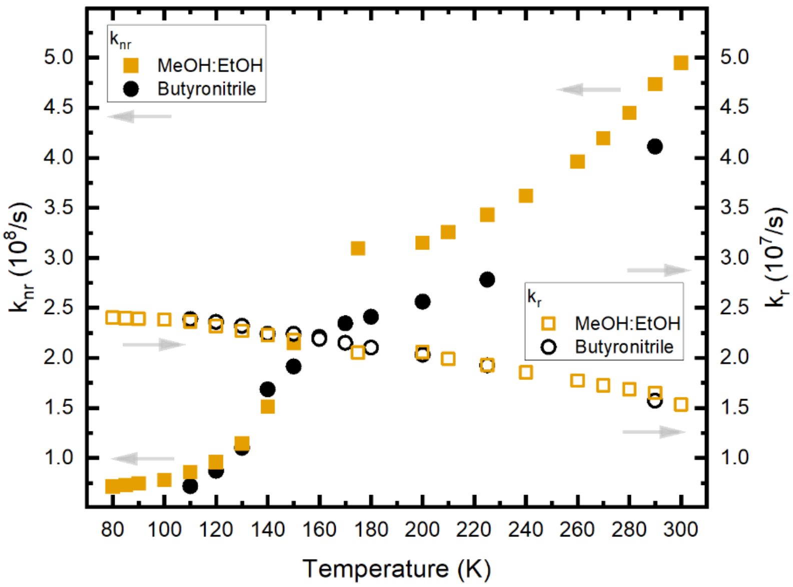

Figure S23. Temperature dependent radiative (open symbols) and non-radiative (closed symbols) recombination rates of [Fe^{III}(phtmeimb)$_2$]PF_6 in MeOH:EtOH (orange squares) and butyronitrile (black circles).

Finally, the calculated temperature dependent emission quantum yield is presented in Figure S24. At RT the quantum yield is found to be ~3 % which is in good agreement with the ~2 % reported previously. [1] It is noteworthy that the quantum yield values presented here are based on new sets of experimental data calculated based on a different formalism, compared to the previously reported value. At 80 K the quantum yield increased to ~25 %. Whereby the major rise appears below the freezing point of the solvent and is mainly related to the increase in luminescence lifetime.

For comparison, the relative emission quantum yield is calculated based on the temperature dependent luminescence intensity measurements (c.f. Figure 4) as well as the temperature dependent absorption. For this, the luminescence intensity is measured at various temperatures with fixed excitation wavelength (500 nm) and excitation density. Then the luminescence intensity is integrated over the whole recorded spectrum. Additionally, the temperature depended absorption spectra are integrated over a ±5 nm window around 500 nm. At 275 K, the integrated luminescence intensity is assumed to correspond to 3 % quantum yield. For the other temperatures the integrated emission intensities are first scaled according to the change in integrated extinction coefficient, in order to normalize them for the number of absorbed photons. Afterwards, the integrated emission intensity is converted to relative quantum yield based on the integrated emission intensity at 275 K. The resulting relative emission quantum yield is in good agreement with the values calculated based on the Strickler-Berg formalism.

In case of the MeOH:EtOH solvent mixture the situation is different. Due to the additional quenching mechanism introduced by the photochemical charge separation (c.f. Figure 4), the luminescence

intensity is no longer directly proportional to the emission quantum yield. Hence, the relative quantum yield cannot be extracted from the luminescence intensity.

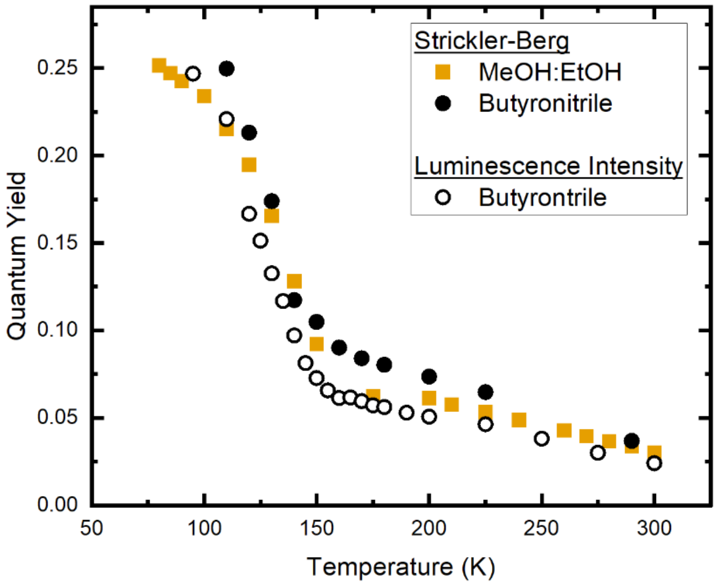

Figure S24. Temperature dependent emission quantum yield of $[Fe^{III}(phtmeimb)_2]PF_6$ in MeOH:EtOH (orange squares) and butyronitrile (black circles) calculated from the extinction coefficient and luminescence lifetime. Additionally, emission quantum yield based on temperature dependent luminescence intensity is show for $[Fe^{III}(phtmeimb)_2]PF_6$ in butyronitrile (black open circles).

Quantum Chemical Calculations

Structure relaxation of $^{2}[Fe^{III}(phtmeimb)_2]^{+}$, $^{4}[Fe^{III}(phtmeimb)_2]^{+}$, $^{3}[Fe^{IV}(phtmeimb)_2]^{+2}$ and $^{1}[Fe^{II}(phtmeimb)_2]$ metal complexes were performed with DFT method using the modified hybrid B3LYP functional with a 15% of HF exchange (B3LYP*) and the D3 Grimme atom-pairwise dispersion correction as implemented in the ORCA-4.2.1 package. [14–17] The def2-TZVP triplet zeta valence with polarization functions together with the def2/J auxiliary basis set was set in all calculations. [18,19] Orca implemented integral grid Grid4 was chosen for the iterative SCF as well as the TightSCF criteria while the Grid5 grid was imposed for the final energy evaluation. RIJCOSX RI approximation was also considered to speed up the calculations. The solvent was described as a continuum by the Conductor-like Polarizable Continuum model CPCM with the dielectric constant of acetonitrile (Ɛ=36.7) or methanol (Ɛ=32.63). Frequency calculation was performed in the relaxed $^{2}[Fe^{III}(phtmeimb)_2]^{+}$ geometry in acetonitrile for predicting the infrared spectrum. $^{2}[Fe(phtmeimb)_2]^{+}$ LMCT excited state minimum was fully optimized with TDDFT using the same settings as for DFT calculations together with the def2-TZVP/C auxiliary basis set, although vacuum was applied during the minimization.

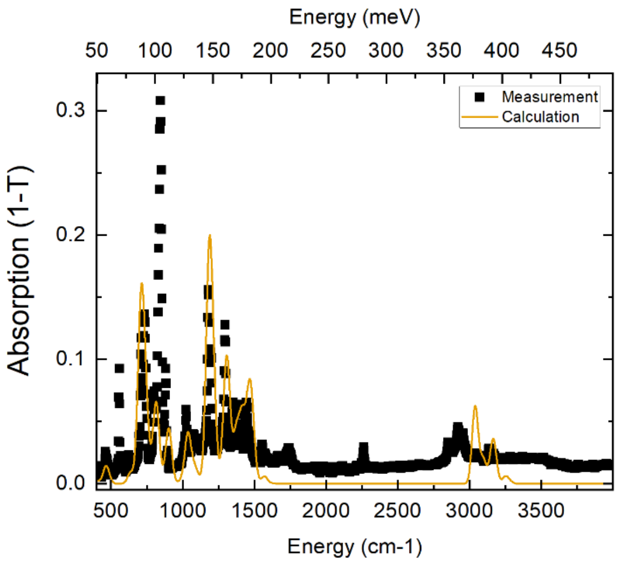

Figure S25. Calculated IR vibrational transition and intensities from DFT (B3LYP*/def2-TZVP/Acetonitrile) compared with the measured IR-spectrum.

An experimental IR spectrum was recorded from the neat compound on a Bruker, Alpha-P FTIR spectrometer (see Figure S25). A quantum chemical frequency calculation was also performed on the optimized ground state geometry and compared with the measured IR spectrum (Figure S25) to explore the vibrational modes involved in the excited state deactivation of $^{2}[Fe^{III}(phtmeimb)_2]^{+}$. Good agreement between experimental and calculated IR spectrum within the 1000-1300 cm^{-1} range of energy ensures confidence in the analysis of all the computed vibrational modes. For completeness, it can be noted that modes with negative frequencies were discarded from further consideration after

identification as spurious low-energy stretching modes. The energy and nature of the frequencies are listed in Table S12. The vibrations in the 1000-1300 cm^{-1} range are mainly characterized by C-H bond bending and stretching in both Imidazol and Phenyl group frames. Interesting stretching involving boron atoms were also identified in some vibrations specially at 1218.52 cm^{-1} and 1219.20 cm^{-1} (171 and 172 vibrational modes). The bond distances of the TD-DFT relaxed ^{2}LMCT (Table S9) show a clear distorted geometry regarding both ligands L_1 and L_2 respect to the $L_{1/2}$ (L_1 and L_2 are equivalent) geometries in the GS. L_1 ligand is farther distorted than L_2 such that B-N_2 bond is 0.03 Å more elongated (the atom numbering is displayed in Figure S26) and Fe-C_2 bond is 0.03 Å shorter respect to the equivalent bond in ligand L_2. B-N_2 and Fe-C_2 bonds are also similarly distorted if compared to the GS $L_{1/2}$, respectively 0.04 Å and 0.03 Å. Such a distortion in the geometry is also reflected in the calculated spin density of the LMCT state (Figure S26), which highlights a non-uniform distribution of the density along the ligands. Since from the temperature dependent steady-state emission vibrational a component in the LMCT state deactivation around 1130 cm^{-1} has been identified, the calculated vibrational frequencies in 1000-1300 cm^{-1} energy range have boron stretching contributions, and finally the LMCT geometry is distorted around B-N bonds, then the vibration involved in the non-radiative decay can be attributed to the boron bonds flexibility.

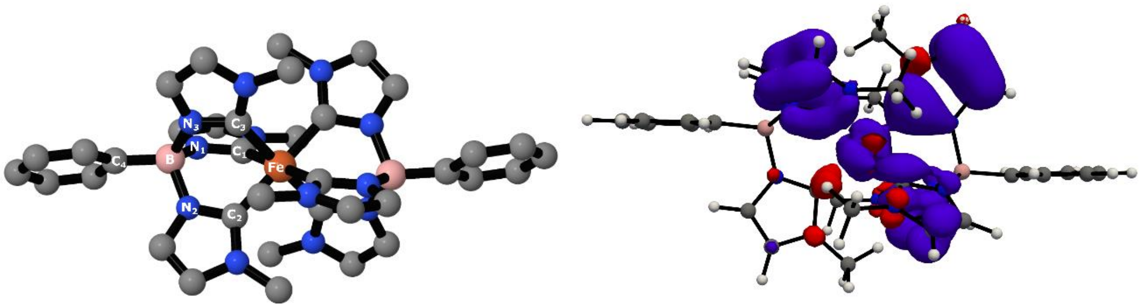

Figure S26. ^{2}[Fe^{III}(phtmeimb)$_2$]$^+$ LMCT geometry with atom labels, hydrogens are hidden for clarity, and corresponding spin density. Distances are displayed in Table SI-DIS.

Table S11. Bond distances in Å for selected bonds for the relaxed ^{2}[Fe^{III}(phtmeimb)$_2$]$^+$ LMCT and GS states, ^{1}[Fe^{II}(phtmeimb)$_2$] GS and ^{3}[Fe^{IV}(phtmeimb)$_2$]$^{+2}$ GS geometries for both ligands L_1 and L_2. Bond labels are depicted in Figure S26.

	^{2}[Fe^{III}(phtmeimb)$_2$]$^+$			^{1}[Fe^{II}(phtmeimb)$_2$]	^{3}[Fe^{IV}(phtmeimb)$_2$]$^{+2}$
**Bond**	FeL_1 ^{LMCT}	FeL_2 ^{LMCT}	$FeL_{1/2}$ ^{GS}	$FeL_{1/2}$ ^{GS}	$FeL_{1/2}$ ^{GS}
**B-N_1**	1.55	1.57	1.57	1.57	1.57
**B-N_2**	1.60	1.57	1.56	1.56	1.57
**B-N_3**	1.55	1.57	1.57	1.57	1.57
**B-C_4**	1.61	1.61	1.60	1.61	1.60
**Fe-C_1**	1.99	2.00	2.00	1.97	2.00
**Fe-C_2**	1.95	1.98	1.98	1.95	1.98
**Fe-C_3**	1.99	2.00	1.99	1.97	2.00
**Fe-B**	3.22	3.20	3.19	3.17	3.18

Table S12. Selected calculated IR vibrational transition frequencies and intensities from DFT (B3LYP*/def2-TZVP/Acetonitrile) in relaxed $^{2}[Fe^{III}(phtmeimb)_2]^{+}$. “im” for Imidazol and “benz” for Benzene.

Vibration	Frequency /cm^{-1}	Intensity	Assignment
**125**	1013.65	0.000233	C-H Bending + B Stretching
**126**	1014.88	1.366940	C-H benz Bending
**127**	1015.15	0.016580	C-H benz/im Bending
**128**	1023.54	0.003936	C-H im Bending
**129**	1024.39	7.660816	C-H im Bending + B Stretching
**130**	1025.94	13.010406	C-H im Bending + B Stretching
**131**	1028.74	0.225362	C-H im Bending
**132**	1030.06	6.156683	C-H im Bending
**133**	1035.84	0.131558	C-H im Bending
**134**	1036.49	63.456100	C-H im Bending
**135**	1036.99	79.443286	C-H benz/im Bending + B Bending
**136**	1037.95	63.987736	C-H im Bending
**137**	1038.57	0.041033	C-H im Bending
**138**	1039.27	0.145896	C-H im Bending
**139**	1058.95	4.938397	C-H benz/im Bending + B Bending
**140**	1059.04	0.855143	C-H benz/im Bending + B Bending
**141**	1071.52	0.028676	C-H im Bending
**142**	1079.98	0.017401	C-H im Bending
**143**	1080.42	25.296220	C-H im Bending
**144**	1083.26	30.539434	C-H im Bending
**145**	1085.17	7.277918	C-H im Bending
**146**	1086.70	0.016771	C-H im Bending
**147**	1091.50	0.045727	C-H im/benz Bending
**148**	1095.98	11.478168	C-H benz Bending
**149**	1096.78	0.127208	C-H im/benz Bending
**150**	1116.62	0.003625	C-H im Bending
**151**	1117.69	0.596547	C-H im Bending
**152**	1124.38	0.999651	C-H im Bending
**153**	1126.95	6.224368	C-H im Bending
**154**	1138.71	0.005333	C-H im Bending
**155**	1139.79	0.001397	C-H im Bending
**156**	1140.30	1.190869	C-H im Bending
**157**	1140.68	0.960301	C-H im Bending
**158**	1141.34	0.062522	C-H im Bending
**159**	1141.67	0.693249	C-H im Bending
**160**	1142.14	0.065392	C-H im Bending
**161**	1163.00	0.008553	C-H benz Bending
**162**	1163.00	0.739545	C-H benz Bending
**163**	1178.41	8.926206	C-H benz Bending +B Stretching
**164**	1179.56	3.919175	C-H benz Bending +B Stretching
**165**	1180.80	417.722616	C-H benz Bending +B Stretching
**166**	1181.22	388.521893	C-H im Bending +B Stretching
**167**	1192.83	1.736021	C-H im Bending
**168**	1194.17	123.829130	C-H im Bending
**169**	1202.29	1.298381	C-H im/benz Bending +B
**170**	1202.64	257.914674	C-H im/benz Bending +B
**171**	1218.52	194.560923	B Stretching + C-H im/benz Bending
**172**	1219.20	0.182441	B Stretching + C-H im/benz Bending

**173**	1268.65	0.001065	C-H Bending + C-N Stretching
**174**	1275.23	17.120862	C-H Bending + C-N Stretching
**175**	1285.07	0.008107	C-H Bending Im + C-N Stretching
**176**	1290.00	98.924001	C-H Bending Im + C-N Stretching +B Stretching

Electronic states landscapes of ^{2}[Fe^{III}(phtmeimb)$_2$]$^+$ were investigated for the fully optimised ^{2}GS and ^{4}MC geometries by single point calculations using the previously described computational settings. Time-dependent DFT (TD-DFT) calculations were performed to identify the quasi-degenerate states in the ^{2}GS as well as the ^{4}MC states, additionally, the ^{2}LMCT excited states (energies available in Table S12). The vertical excitations calculated from the open-shell systems were validated with the available ^{2}[Fe^{III}(phtmeimb)$_2$]$^+$ visible absorption and emission spectral data. [1] A linear response of the TD-DFT ^{2}[Fe^{III}(phtmeimb)$_2$]$^+$ LMCT energy and the uDFT ^{4}MC states with the HF exchange percentage where identified in Figure S27. The predicted ^{2}LMCT absorption band is red shifted with the reduction of the HF exchange amount whilst the ^{4}MC state energy shows an inverse energy response. The final employed HF exchange for the description of the electronic structure and final energy was decided based on the fitting with the experimental evidences. Therefore, 12 % HF exchange was chosen for predicting an absorption maximum peak at 2.51 eV (2.47 eV reported in the literature) and a ^{2}LMCT emission at 1.83 eV (1.89 eV reported in the literature). All validation points were performed in acetonitrile and the final energies were recalculated in methanol with B3LYP(12%)-D3/def2-TZVP/PCM(Methanol)//B3LYP*-D3/def2-TZVP/PCM(Acetonitrile) level of theory.

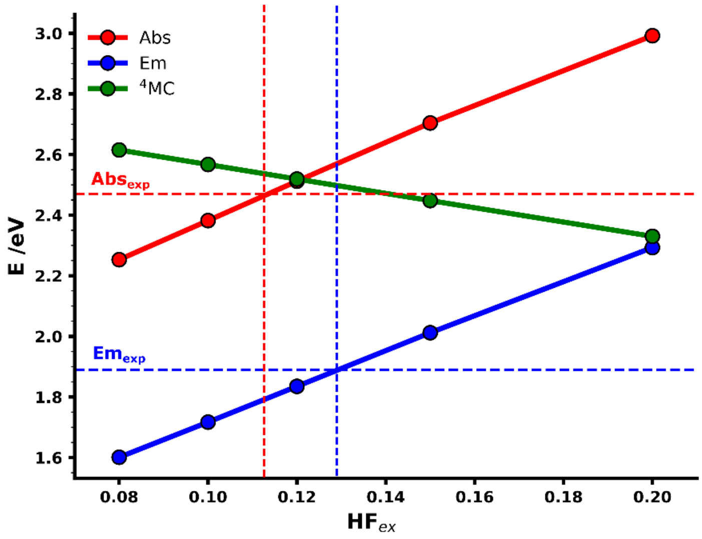

Figure S27. Energy response of uDFT and open-shell TD-DFT with the amount of HF exchange in the optimised ^{2}[Fe^{III}(phtmeimb)$_2$]$^+$ GS state at B3LYP*-D3/def2-TZVP/PCM(Acetonitrile). ^{2}[Fe^{III}(phtmeimb)$_2$]$^+$ LMCT excited state energy displayed in bold red and ^{4}[Fe^{III}(phtmeimb)$_2$]$^+$ MC state energy in bold green. Emission energy from the minimum ^{2}[Fe(phtmeimb)$_2$]$^+$ LMCT was also calculated for the five different percentages of HF exchange and displayed in bold blue. The reported experimental values for the maximum of the absorption band (red) and emission maxima peak (blue) are represented in dashed lines.

The photoinduced disproportionation process in $^{2}[Fe^{III}(phtmeimb)_2]^{+}$ was investigated by assuming two-non-interacting bodies as an advantage on the characterization of the charge separated (CS) states, since $[^{1}Fe^{II}|^{3}Fe^{IV}]$ system have different spin with the corresponding $[^{2}Fe^{III}|^{2}Fe^{III}]$ GS unlike the interacting $^{3}[Fe^{II}Fe^{IV}]$ system with its $^{3}[^{2}Fe^{III}]$ GS. Thus, energetics of the bimolecular system in the GS, LMCT and CS minimum states were calculated by energy addition of the individual molecular units, $[^{2}Fe^{III}|^{2}Fe^{III}]$, $[^{2}Fe^{III}*|^{2}Fe^{III}]$ and $[^{1}Fe^{II}|^{3}Fe^{IV}]$ and $[^{3}Fe^{IV}|^{1}Fe^{II}]$ respectively. We have considered CS mechanisms by both, photoreduction and photoxidation of the $^{2}[Fe^{III}(phtmeimb)_2]^{+}$ LMCT excited state, returning the $[^{1}Fe^{II}|^{3}Fe^{IV}]$ and $[^{3}Fe^{IV}|^{1}Fe^{II}]$ states. Single point calculations were performed over the relaxed structures to calculate the corresponding lower-lying $[^{2}Fe^{III}|^{2}Fe^{III}]$ GS and $[^{1}Fe^{II}|^{3}Fe^{IV}]$ and $[^{3}Fe^{IV}|^{1}Fe^{II}]$ CS state. Localization of the two almost degenerate $[^{2}Fe^{III}*|^{2}Fe^{III}]$ GS, $[^{2}Fe^{III}*|^{2}Fe^{III}]$ LMCT and $[^{1}Fe^{II}|^{3}Fe^{IV}*]$ and $[^{3}Fe^{IV}*|^{1}Fe(II)]$) excited states were computed from TD-DFT calculations on $^{2}[Fe^{III}(phtmeimb)_2]^{+}$ and $^{3}[Fe^{IV}(phtmeimb)_2]^{+2}$ geometries. All the computed energies are listed in Table S13. Electron transitions in $^{3}[Fe^{IV}(phtmeimb)_2]^{+2}$ metal complex were assessed with the available experimental absorption spectra (LMCT band identified at 1.53 eV (810 nm) and TD-DFT prediction of 1.47 eV). [1] $[^{1}Fe^{II}*|^{3}Fe^{IV}]$ excited states were excluded in this study for expecting these states to appear at higher energies than the $[^{1}Fe^{II}|^{3}Fe^{IV}*]$ excited states, according to the spectroelectrochemistry studies on $[Fe^{II}(phtmeimb)_2]^{+}$. [1] Intense absorption bands of $^{3}[Fe^{IV}(phtmeimb)_2]^{+2}$ were identified in the IR range (810 nm) of the spectra while $^{1}[Fe(phtmeimb)_2]$ first absorption band lies in the visible range (502 nm).

Table S13. Doublet and quartet state energies (eV) relative to the ^{2}GS in the $^{2}[Fe^{III}(phtmeimb)_2]^{+}$ and $^{4}[Fe^{III}(phtmeimb)_2]^{+}$ relaxed geometries. Calculations at the B3LYP(12%)-D3/def2-TZVP/PCM(Methanol)//B3LYP*-D3/def2-TZVP/PCM(Acetonitrile) level of theory.

State	$^{2}[Fe^{III}(phtmeimb)_2]^{+}$	$^{4}[Fe^{III}(phtmeimb)_2]^{+}$
$^{2}[Fe^{III}(phtmeimb)_2]^{+}$GS	0.000	0.626
$^{2}[Fe^{III}(phtmeimb)_2]^{+}$MC	0.243	0.919
$^{2}[Fe^{III}(phtmeimb)_2]^{+}$MC	0.362	1.125
$^{2}[Fe^{III}(phtmeimb)_2]^{+}$LMCT	2.388	2.156
$^{2}[Fe^{III}(phtmeimb)_2]^{+}$LMCT	2.443	2.281
$^{2}[Fe^{III}(phtmeimb)_2]^{+}$LMCT	2.512	2.857
$^{4}[Fe^{III}(phtmeimb)_2]^{+}$MC	2.519	1.429

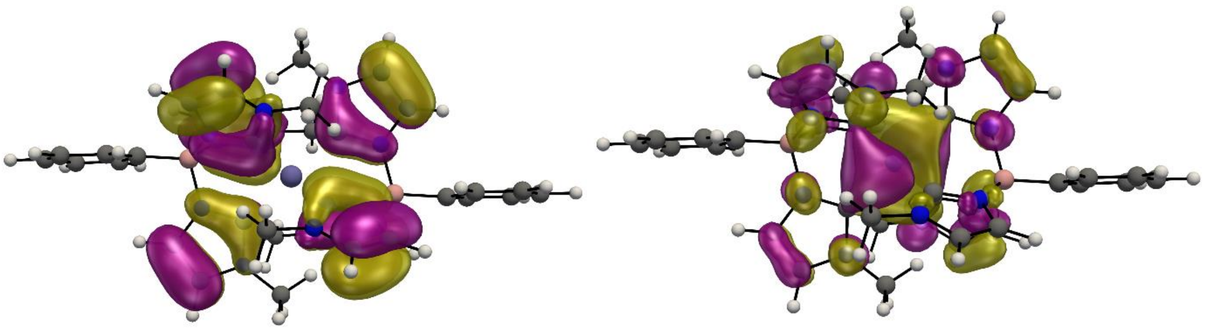

Figure S28. Molecular orbitals involved in the main electronic transition (184b → 187b) corresponding to the lower lying ^{2}LMCT excited state at the $^{2}[Fe^{III}(phtmeimb)^{2}]^{+}$ GS geometry. Left orbital is a π ligand orbital (184b) and right orbital is a d orbital (187).

Table S14. State energies in eV in the [Fe^{III}*|Fe^{III}], [Fe^{III}|Fe^{III}] and [Fe^{IV}|Fe^{II}] relaxed structures relative to the [Fe^{III}|Fe^{III}] GS energy. Calculations at the B3LYP(12 %)-D3/def2-TZVP/PCM(Methanol)//B3LYP*-D3/def2-TZVP/PCM(Acetonitrile) level of theory.

State	[Fe^{III}*|Fe^{III}]	[Fe^{III}|Fe^{III}]	[Fe^{IV}|Fe^{II}]
**[FeIII|FeIII]**	0.212	0.000	0.134
**[FeIII^{MC}|FeIII]**	0.403	0.243	0.361
**[FeIII^{MC}|FeIII]**	0.588	0.362	0.465
**[FeIII^{LMCT}|FeIII]**	2.304	2.388	2.545
**[FeIII|FeIII^{MC}]**	0.455	-	0.336
**[FeIII|FeIII^{MC}]**	0.574	-	0.484
**[FeIII|FeIII^{LMCT}]**	2.600	-	2.319
**[FeIV|FeII]**	1.795	1.582	1.430
**[FeIV$^{MC\|}$FeII]**	2.323	2.086	1.922
**[FeIV$^{MC\|}$FeII]**	2.384	2.090	1.923
**[FeII|FeIV]**	1.702	-	-
**[FeII|FeIV^{MC}]**	2.206	-	-
**[FeII|FeIV^{MC}]**	2.210	-	-

Figure S29. Spin density plots of the relaxed structures ^{2}[Fe^{III}(phtmeimb)$_2$]$^{+}$ (left) and ^{3}[Fe^{IV}(phtmeimb)$_2$]$^{+2}$ (right).

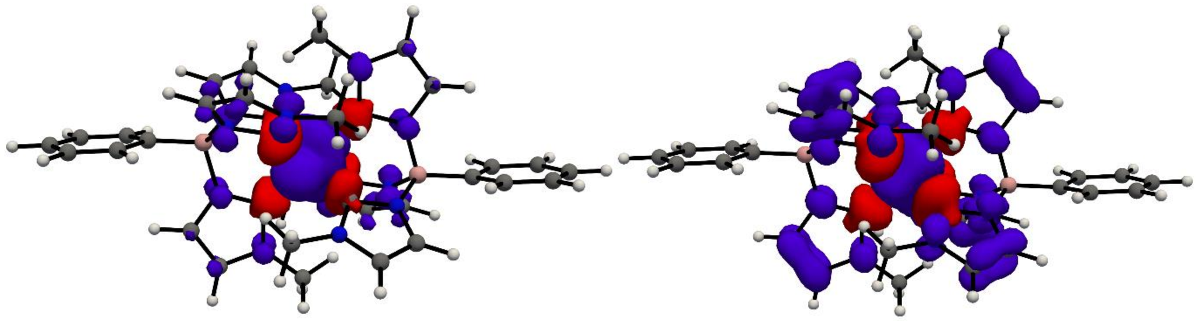

Table S15. TD-DFT vertical excitations energies, wavelength, oscillator strengths, electron transitions and coefficients and state assignments for $^{2}[Fe^{III}(phtmeimb)_2]^{+}$, $^{4}[Fe^{III}(phtmeimb)_2]^{+}$ and $^{3}[Fe^{IV}(phtmeimb)_2]^{+2}$ in relaxed $^{2}[Fe^{III}(phtmeimb)_2]^{+}$ GS geometry.

$^{2}[Fe^{III}(phtmeimb)_2]^{+}$

Energy /eV	Fosc	Electron transition				
1	5098.5	0.243	0.000	186b → 187b	0.914	MC
2	3419.4	0.363	0.000	185b → 187b	0.945	MC
3	519.3	2.388	0.000	184b → 187b	0.992	LMCT
4	507.5	2.443	0.003	182b → 187b<br>183b → 187b	0.493<br>0.491	LMCT
5	493.5	2.512	0.006	182b → 187b<br>183b → 187b	0.494<br>0.485	LMCT
6	471.6	2.629	0.005	181b → 187b	0.985	LMCT

$^{4}[Fe^{III}(phtmeimb)_2]^{+}$

State	Wavelength /nm	Energy /eV				
1	3164.6	0.392	0.000	185b → 186b	0.716	MC
2	2573.6	0.482	0.000	185b → 187b	0.852	MC
3	2312.4	0.536	0.000	188a → 191a	0.709	MC
4	1123.7	1.103	0.000	188a → 189a	0.793	MLCT
5	1179.3	1.051	0.0296	188a → 190a	0.962	MLCT
6	1010.6	1.227	0.000	188a → 193a	0.700	MLCT

$^{3}[Fe^{IV}(phtmeimb)_2]^{+2}$

State	Wavelength /nm	Energy /eV				
1	2462.4	0.504	0.000	177b → 186b<br>177b → 187b	0.667<br>0.300	MC
2	2441.8	0.508	0.000	177b → 187b<br>177b → 186b	0.667<br>0.300	MC
3	887.3	1.397	0.000	185b → 186b	0.987	LMCT
4	869.9	1.425	0.000	185b → 187b	0.988	LMCT
5	844.9	1.467	0.015	184b → 186b	0.677	LMCT
6	823.9	1.505	0.017	183b → 186b<br>184b → 187b	0.476<br>0.417	LMCT

TableS16. TD-DFT vertical excitations energies, wavelength, oscillator strengths, electron transitions and coefficients and state assignments for $^{2}[Fe^{III}(phtmeimb)_2]^{+}$ and $^{3}[Fe^{IV}(phtmeimb)_2]^{+2}$ in the $^{2}[Fe^{III}(phtmeimb)_2]^{+}$ LMCT geometry.

	**$^{2}[Fe^{III}(phtmeimb)_2]^{+}$**					
State	Wavelength /nm	Energy /eV	Fosc	Electron transition	Coefficient	Assignment
1	6501.2	0.191	0.000	186b → 187b	0.878	MC
2	3297.0	0.376	0.000	185b → 187b	0.911	MC
3	593.1	2.092	0.029	184b → 187b	0.981	LMCT
4	529.4	2.344	0.014	182b → 187b	0.985	LMCT
5	525.1	2.361	0.060	183b → 187b	0.808	LMCT
6	512.3	2.420	0.040	181b → 187b	0.816	LMCT
	**$^{3}[Fe^{IV}(phtmeimb)_2]^{+2}$**					
State	*Wavelength /nm*	*Energy /eV*				
1	4262.2	0.528	0.000	177b → 186b	0.948	MC
2	4745.3	0.588	0.000	177b → 187b	0.948	MC
3	8539.5	1.059	0.000	185b → 186b	0.917	LMCT
4	10422.3	1.292	0.012	185b → 187b	0.912	LMCT
5	10824.6	1.342	0.000	184b → 186b	0.919	LMCT
6	11945.0	1.481	0.000	182b → 187b	0.786	LMCT

Table S17. TD-DFT vertical excitations energies, wavelength, oscillator strengths, electron transitions and coefficients and state assignments for $^{2}[Fe^{III}(phtmeimb)_2]^{+}$ in the relaxed $^{1}[Fe^{III}(phtmeimb)_2]^{+}$ geometry.

	**$^{1}[Fe^{III}(phtmeimb)_2]^{+}$**					
State	Wavelength /nm	Energy /eV	Fosc	Electron transition	Coefficient	Assignment
1	6111.1	0.203	0.000	186b → 187b	0.919	MC
2	3534.9	0.351	0.000	185b → 187b	0.951	MC
3	567.2	2.186	0.000	184b → 187b	0.991	LMCT
4	558.8	2.219	0.023	183b → 187b	0.990	LMCT
5	525.3	2.360	0.068	182b → 187b	0.978	LMCT
6	505.0	2.455	0.062	181b → 187b	0.985	LMCT

Table S18. TD-DFT vertical excitations energies, wavelength, oscillator strengths, electron transitions and coefficients and state assignments for $^{3}[Fe^{IV}(phtmeimb)_2]^{+2}$ and $^{2}[Fe^{II}(phtmeimb)_2]^{+}$ in the relaxed $^{3}[Fe^{IV}(phtmeimb)_2]^{+2}$ geometry.

State	Wavelength /nm	Energy /eV	Fosc	Electron transition	Coefficient	Assignment
	**$^{3}[Fe^{IV}(phtmeimb)_2]^{+2}$**					
1	2523.5	0.491	0.000	177b → 187b	0.961	MC
2	2514.5	0.493	0.000	177b → 186b	0.960	MC
3	863.7	1.436	0.000	185b → 186b	0.989	LMCT
4	848.5	1.461	0.000	185b → 187b	0.988	LMCT
5	808.9	1.533	0.009	183b → 186b	0.783	LMCT
6	789.4	1.571	0.030	182b → 186b<br>183b → 187b<br>184b → 186b	0.403<br>0.334<br>0.232	LMCT
	**$^{2}[Fe^{III}(phtmeimb)_2]^{+}$**					
1	5461.1	0.227	0.000	186b → 187b	0.920	MC
2	3742.6	0.331	0.000	185b → 187b	0.953	MC
3	514.2	2.411	0.000	184b → 187b	0.990	LMCT
4	511.6	2.424	0.012	183b → 187b	0.988	LMCT
5	491.9	2.521	0.061	182b → 187b	0.975	LMCT
6	464.2	2.671	0.033	181b → 187b	0.987	LMCT

Table S19. TD-DFT vertical excitations energies, wavelength, oscillator strengths, electron transitions and coefficients and state assignments for $^{4}[Fe^{III}(phtmeimb)_2]^+$ and $^{2}[Fe^{III}(phtmeimb)_2]^+$ in the relaxed $^{4}[Fe^{III}(phtmeimb)_2]^+$ geometry.

$^{4}[Fe^{III}(phtmeimb)_2]^+$

State	Wavelength /nm	Energy /eV	Fosc	Electron transition	Coefficient	Assignment
1	2665.9	0.465	0.000	185b → 186b	0.868	MC
2	2479.6	0.500	0.000	185b → 187b	0.869	MC
3	697.7	1.777	0.000	188a → 189a	0.976	LMCT
4	483.7	2.563	0.017	183b → 186b	0.890	LMCT
5	480.3	2.581	0.000	184b → 186b<br>185b → 188b	0.609<br>0.321	LMCT
6	476.9	2.600	0.000	185b → 188b<br>184b → 186b	0.523<br>0.361	LMCT

$^{2}[Fe^{III}(phtmeimb)_2]^+$

State	*Wavelength /nm*	*Energy /eV*	*Fosc*	*Electron transition*	*Coeffi*	
1	4222.7	0.294	0.000	186b → 187b	0.881	MC
2	2483.0	0.499	0.000	185b → 187b	0.940	MC
3	809.8	1.531	0.000	186b → 188b<br>187a → 188a	0.395<br>0.255	MC
4	748.7	1.656	0.000	185b → 188b<br>183a → 188a	0.446<br>0.248	MC
5	555.5	2.232	0.000	187a → 188a<br>183a → 188a	0.288<br>0.262	MC
6	473.6	2.618	0.000	184b → 187b<br>183a → 188a	0.278<br>0.228	LMCT

References

1 K. S. Kjær, N. Kaul, O. Prakash, P. Chábera, N. W. Rosemann, A. Honarfar, O. Gordivska, L. A. Fredin, K. Bergquist, L. Häggström, T. Ericsson, L. Lindh, A. Yartsev, S. Styring, P. Huang, J. Uhlig, J. Bendix, D. Strand, V. Sundström, P. Persson, R. Lomoth and K. Wärnmark, *Science (1979)*, , DOI:10.1126/science.aau7160.

2 A. v Lesikar, *J Solution Chem*, 1977, **6**, 81–93.

3 M. A. Ramos, B. Kabtoul and M. Hassaine, *Philosophical Magazine*, 2011, **91**, 1847–1856.

4 H. Nakayama, K. Omori, K. Ino-u-e and K. Ishii, *J Phys Chem B*, 2013, **117**, 10311–10319.

5 C. Reichardt, *Solvents and Solvent Effects in Organic Chemistry*, WILEY-VCH, 2003.

6 J. Mooney and P. Kambhampati, *J Phys Chem Lett*, 2013, **4**, 3316–3318.

7 M. Abrahamsson, H.-C. Becker and L. Hammarström, *Dalton Transactions*, 2017, **46**, 13314–13321.

8 J. v. Caspar and T. J. Meyer, *Inorg Chem*, 1983, **22**, 2444–2453.

9 J. v Caspar and T. J. Meyer, *Application of the Energy Gap Law to Nonradiative, Excited-State Decay*, 1983, vol. 87.

10 A. Juris, V. Balzani, F. Barigelletti, S. Campagna, P. Belser and A. von Zelewsky, *Coord Chem Rev*, 1988, **84**, 85–277.

11 M. Abrahamsson, H. Wolpher, O. Johansson, J. Larsson, M. Kritikos, L. Eriksson, P.-O. Norrby, J. Bergquist, L. Sun, B. Åkermark and L. Hammarström, *Inorg Chem*, 2005, **44**, 3215–3225.

12 M. Abrahamsson, H.-C. Becker and L. Hammarström, *Dalton Transactions*, 2017, **46**, 13314–13321.

13 S. J. Strickler and R. A. Berg, *J Chem Phys*, 1962, **37**, 814–822.

14 F. Neese, F. Wennmohs, U. Becker and C. Riplinger, *J Chem Phys*, 2020, **152**, 224108.

15 S. Grimme, S. Ehrlich and L. Goerigk, *J Comput Chem*, 2011, **32**, 1456–1465.

16 A. D. Becke, *J Chem Phys*, 1993, **98**, 5648–5652.

17 M. Reiher, O. Salomon and B. Artur Hess, *Theoretical Chemistry Accounts: Theory, Computation, and Modeling (Theoretica Chimica Acta)*, 2001, **107**, 48–55.

18 F. Weigend and R. Ahlrichs, *Physical Chemistry Chemical Physics*, 2005, **7**, 3297.

19 F. Weigend, *Physical Chemistry Chemical Physics*, 2006, **8**, 1057.
